# Hydrogenation of Uracil in Solid *Para*-Hydrogen: Infrared Spectra of Monohydrogenated Radicals (5*H*‑C_4_H_5_N_2_O_2_, 6*H*‑C_4_H_5_N_2_O_2_, and 4*OH*-C_4_H_5_N_2_O_2_) and Their Implications for RNA-Related Chemistry in the Interstellar Medium

**DOI:** 10.1021/acs.jpca.6c05066

**Published:** 2026-10-20

**Authors:** Prasad Ramesh Joshi, Manish Kumar Tripathi, Yuan-Pern Lee

**Affiliations:** † Department of Applied Chemistry and Institute of Molecular Science, 34914 National Yang Ming Chiao Tung University, Hsinchu 300093, Taiwan; ‡ Center for Emergent Functional Matter Science, National Yang Ming Chiao Tung University, Hsinchu 300093, Taiwan

## Abstract

Uracil (C_4_H_4_N_2_O_2_), an essential RNA nucleobase central to the RNA World hypothesis and the prebiotic emergence of life, has been detected in pristine asteroid samples, yet its survival mechanism in nonenergetic interstellar environments remains an elusive paradox. Backed by high-level CCSD­(T)//B3LYP quantum-chemical calculations, we successfully captured and characterized the IR spectra of three transient, monohydrogenated radicals for the first time: the dominant 5*H*-C_4_H_5_N_2_O_2_ **(1)** and minor 6*H*-C_4_H_5_N_2_O_2_ **(2)** radicals, resulting from H-addition to the C=C moiety, alongside minor features of the 4-hydroxy-uracil radical 4*OH*-C_4_H_5_N_2_O_2_ **(3)**, resulting from H-addition to the carbonyl oxygen at the C4 position, under hydrogen-rich conditions. Under H-rich conditions, the formation of minor features of the dihydrogenated product 5,6-dihydrouracil (DHU), resulting via secondary H-addition to **(1)** or **(2)**, was also observed. The assignments for **(3)** and DHU are tentative because only a limited number of bands were observed due to low intensities and interference. Crucially, real-time temporal profiling uncovered a striking, counterintuitive recycling mechanism: while both secondary H-addition (forming DHU) and secondary H-abstraction (regenerating uracil) are barrierless, the radical-to-parent regeneration pathway is significantly favored. This unexpected preference suggests that repeated collisions with cosmic hydrogen atoms might not efficiently destroy uracil, thereby drastically extending its chemical lifetime, which might have implications for how uracil could survive and be exogenously seeded onto young planets.

## Graphical abstract

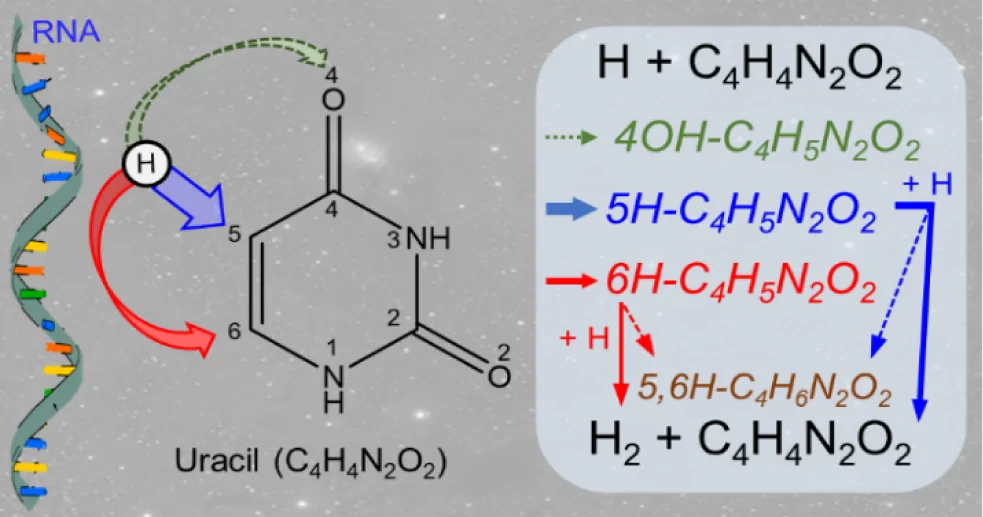

## 1 Introduction

Nucleobases are the fundamental building blocks of ribonucleic acid (RNA) and deoxyribonucleic acid (DNA), which are essential for the genetic, hereditary, and metabolic functions of terrestrial life.^1–3^ Their potential exogenous delivery to the early Earth has long been considered a plausible contributor to the origin of life.^4^ Although nucleobases are hypothesized to exist in interstellar environments, they have yet to be unambiguously detected despite extensive astronomical searches.^5,6^

Uracil is a pyrimidine nucleobase found exclusively in RNA. It is a heterocyclic compound comprising a six-membered aromatic ring with four carbon atoms and two nitrogen atoms (sites N1 and N3). Two exocyclic carbonyl oxygen atoms are attached to the carbons at the C2 and C4 positions; the structure and the atomic numbering of the ring are illustrated in Figure 1. The exocyclic oxygen atoms are numbered according to the ring carbons to which they are attached (e.g., the oxygen bonded to C2 is designated as O2). Uracil has been identified in the Murchison, Murray, and Orgueil carbonaceous chondrites,^7–9^ and its nonterrestrial origin was further confirmed through carbon-isotope measurements in the Murchison meteorite.^10^ More recently, uracil was conclusively detected in pristine samples from the carbonaceous asteroid (162173) Ryugu, reinforcing the theory that such prebiotic molecules are common in carbonaceous asteroids and may be delivered to early Earth.^11^

**Figure 1:**
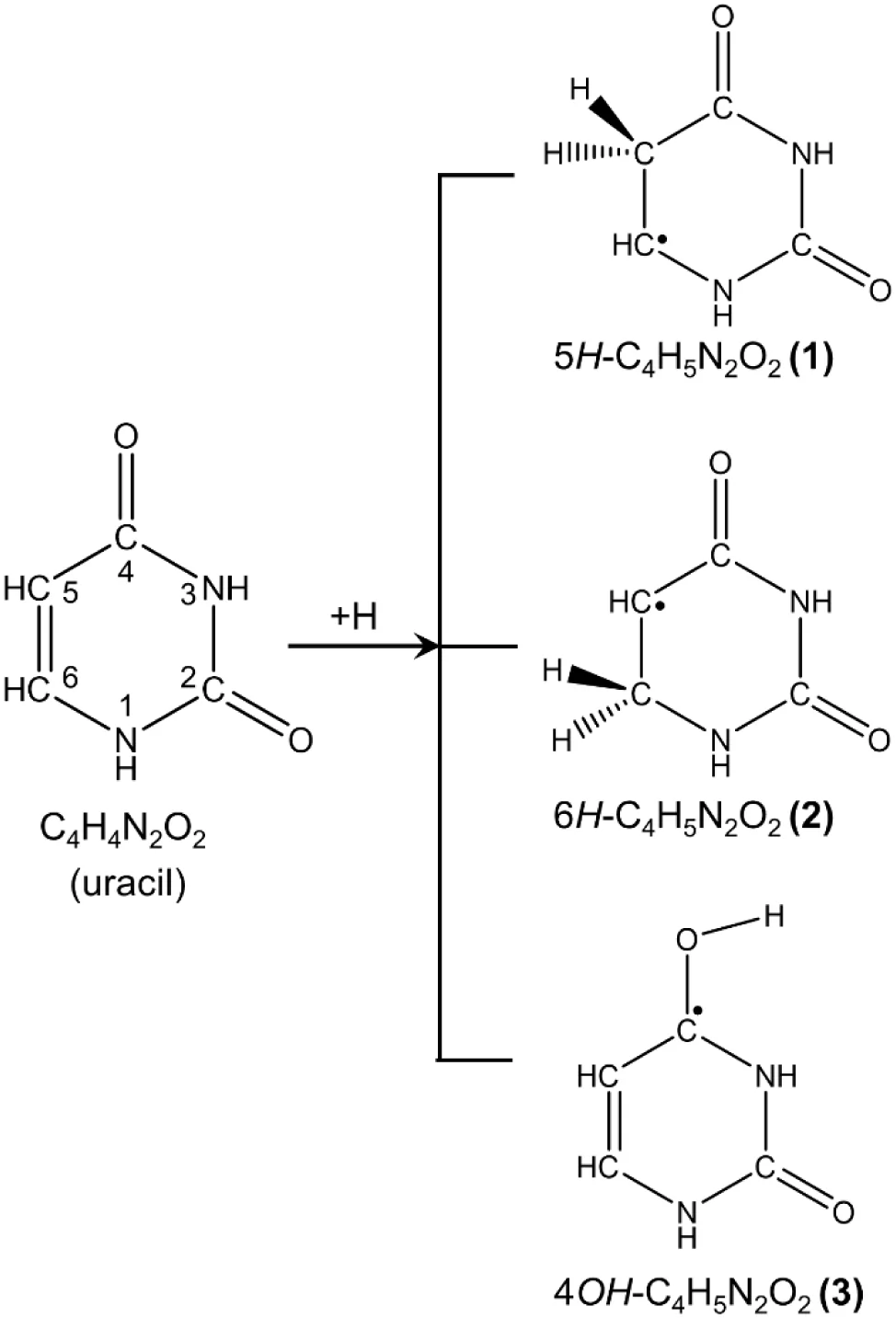
Reaction schemes for the observed reactions of uracil with a hydrogen atom.

To elucidate the abiotic synthesis of this essential molecule, uracil has been extensively investigated in laboratory simulations of extraterrestrial conditions.^12^ In the gas phase, its formation has been reported following high-energy proton irradiation of mixtures containing CO_2_, CO, N_2_, and H_2_O at 297 K.^13–15^ Abiotic formation of several nucleobases, including uracil, has also been observed from formamide condensed onto cosmic-dust analogues, TiO_2_ surfaces, and mineral catalysts under proton irradiation.^16–18^ Theoretically, gas-phase reactions between dehydrogenated formamide and vinyl cyanide have been proposed to yield uracil via a 1H-pyrimidin-2-one (C_4_H_4_N_2_O) intermediate, which subsequently reacts with OH radicals to yield uracil.^19^ Pyrimidine, another key precursor, yields uracil when mixed with pure water ice and irradiated with ultraviolet (UV) light;^20–22^ the reaction mechanism was explored using quantum-chemical computations.^23^ Furthermore, uracil synthesis has been demonstrated from urea solutions subjected to spark discharge or UV irradiation in various atmospheres, including CH_4_/N_2_/H_2_ and acetylene.^24,25^ The formation of uracil, along with other nucleobases, was achieved through the UV irradiation of complex interstellar ice analogues, including H_2_O:CO:NH_3_:CH_3_OH (5:2:2:2) at 10 K and H_2_O:NH_3_:^13^CH_3_OH (2:1:1) at 77 K, followed by thermal processes, mimicking the chemical evolution occurring during star formation in molecular clouds.^26,27^

Radiative and oxidative damage to DNA/RNA often involves reactions with nucleobases via two primary mechanisms: the direct addition of small radicals (such as OH and H, produced by radiolysis of water or other chemical reactions) or electron capture followed by protonation. The reaction of H atoms with uracil is predicted to proceed via three low-energy channels: addition to the C5, C6, or the O atom at the C4 site:^28^

$$H+\mathrm{uracil}\to {5H‐C}_{4}H_{5}N_{2}O_{2}\left(1\right)$$ 1

$$H+\mathrm{uracil}\to {6H‐C}_{4}H_{5}N_{2}O_{2}\left(2\right)$$ 2

$$H+\mathrm{uracil}\to {4\mathrm{OH}‐C}_{4}H_{5}N_{2}O_{2}\left(3\right)$$ 3

as illustrated in Figure 1. Among these products, 5-hydrouracil-6-yl radical (or hexahydro-2,4-dioxo-6-pyrimidinyl, denoted 5*H*-C_4_H_5_N_2_O_2_ or **(1)**) is the most stable, 6-hydrouracil-5-yl (or hexahydro-2,4-dioxo-5-pyrimidinyl radical, denoted 6*H*-C_4_H_5_N_2_O_2_ or **(2)**) and 1,3,5,6-tetrahydro-4-hydroxy-2-oxo-4-pyrimidinyl radical (denoted 4*OH*-C_4_H_5_N_2_O_2_ or **(3)**) are higher in energy by ∼13 and 40 kJ mol^–1^, respectively.

Tureček and coworkers prepared gaseous radicals **(1)** and **(3)** from uracil by either collisional electron transfer with CH_3_SSCH_3_ or femtosecond collisional electron transfer to its cationic precursor, and detected them using neutralization-reionization mass spectrometry.^29,30^ However, these gas-phase experiments differ significantly from conditions in the interstellar medium (ISM). Investigating the reactions of nucleobases, such as uracil, at cryogenic temperatures is essential for understanding their abiotic synthesis and degradation in nonenergetic cosmic environments.

Hydrogen-atom reactions are prevalent in dark molecular clouds where cosmic radiation is attenuated. In our laboratory, we use solid *para*-hydrogen (*p*-H_2_) as a matrix host. This quantum solid offers unique advantages, including diminished cage effects and efficient hydrogen reactions enabled by the chemical diffusion of H atoms.^31^ While *p*-H_2_ is not a direct proxy for interstellar environments, it serves as an effective medium for investigating fundamental reaction pathways relevant to the formation of complex organic molecules (COMs) in the dark interstellar medium. Using this approach, we and others (notably the Tarczay group) have systematically investigated H atom reactions with a wide array of astrochemically relevant species, including alcohols, amides, organic acids, and sugars.^32–49^ A mini-review^50^ and a perspective article^51^ on this subject are also available.

In the present study, we investigated the H atom reaction with uracil in solid *p*-H_2_ at 3.2 K. We successfully identified the IR spectra of the hydrogen-addition products 5*H*-C_4_H_5_N_2_O_2_ **(1)**, 6*H*-C_4_H_5_N_2_O_2_ **(2)**, and 4*OH*-C_4_H_5_N_2_O_2_ **(3)** radicals. Under H-rich conditions, although weak features of the dihydrogenated product 5,6-dihydrouracil (DHU) resulting from secondary H-addition to **(1)** or **(2)** were also observed, the anticorrelation between uracil and radicals **(1)**/**(2)** indicates that the uracil-regeneration pathway via H-abstraction of **(1)** or **(2)** is significantly favored. This unexpected preference may have profound implications for astrochemistry, implying that uracil may survive in harsh H-rich interstellar environments prior to planetary seeding.

## 2 Experimental Method

The experimental technique used to investigate radical intermediates in solid *p*-H_2_ involves a matrix-isolation system coupled with IR absorption spectroscopy, as described in detail elsewhere.^31,52,53^ The cryogenic matrix substrate, a nickel-coated copper plate maintained at 3.2 K using a closed-cycle helium refrigerator, functions as both a deposition surface and a mirror for reflective absorption measurements. IR spectra were recorded using a Fourier-transform infrared (FTIR) spectrometer equipped with a KBr beam splitter and an Hg–Cd–Te (MCT) detector (77 K). Typically, 400 scans at a spectral resolution of 0.25 cm^–1^ were acquired over the spectral region of 550–4000 cm^–1^ at each experimental stage. To prevent unwanted reactions between vibrationally excited H_2_ and Cl atoms during data acquisition, an IR filter with a cutoff at 2.4 μm was employed to block light with wavenumbers >4000 cm^–1^ from the spectrometer’s IR source.

Gaseous uracil was introduced by flowing *p-*H_2_ through an oven containing solid uracil powder (Merck, purity >98%) maintained at ∼387 K. A gaseous mixture of Cl_2_ in *p-*H_2_ was codeposited through a separate gas line. The resulting gaseous mixture of uracil/Cl_2_/*p*-H_2_ (∼1/20/10000) was deposited onto the cold substrate over 6 h at a total flow rate of ∼6 STP cm^3^ min^–1^ (STP defined as 273 K and 760 Torr). *p*-H_2_ was prepared by passing normal H_2_ (99.9999%) through a converter containing an iron­(III) oxide catalyst maintained at 12.9 K using a separate closed-cycle helium refrigerator.

Following deposition, the matrix was subjected to UV photolysis at 365 ± 5 nm for 30 min using a light-emitting diode (LED, 5 W) to generate Cl atoms. Subsequently, the matrix was irradiated with IR light from an external source for 1 h to promote the reaction Cl + H_2_ (*v* = 1) → HCl + H, thereby producing H atoms that can diffuse efficiently through the matrix via the successive formation and breaking of neighboring H–H bonds. After IR irradiation, the matrix was kept in darkness for 10 h to allow H atom tunneling reactions to proceed. The matrix was then subjected to secondary photolysis at 460 and 399 nm for 20 min each using a tunable OPO laser (EKSPLA, NT340; repetition rate 10 Hz, pulse duration 5 ns, ∼1.5 mJ) to categorize the newly observed IR features into distinct groups according to their photochemical behavior. The wavelengths employed for the secondary photolysis were selected based on absorption wavelengths predicted by TD-DFT calculations. Mixing ratios for each species were estimated from the observed absorbance using the method of Tam and Fajardo,^54^ utilizing quantum-chemically predicted IR intensities and the determined optical path length, as detailed in Supporting Information Note 1.

## 3 Computational Method

All quantum-chemical calculations were performed using the Gaussian 16 program suite.^55^ Optimized geometries, vibrational wavenumbers, and IR intensities were computed using density functional theory (DFT) with the B3LYP functional and the Dunning correlation-consistent basis set augmented with diffuse functions (aug-cc-pVTZ).^56^ Subsequently, single-point energies were predicted using the CCSD­(T) method, coupled-cluster with single, double, and perturbative triple excitations, based on the B3LYP/aug-cc-pVTZ-optimized geometries. Zero-point vibrational energies (ZPVE) were corrected using harmonic vibrational wavenumbers calculated at the B3LYP/aug-cc-pVTZ level. The average absolute deviation between experimental values and harmonic vibrational wavenumbers of uracil is 38 ± 32 cm^–1^. To improve the reliability of vibrational assignments, harmonic vibrational wavenumbers were scaled using two distinct linear relationships: *y* = (0.9987 ± 0.0099)*x* – (10.8 ± 12.6) and *y* = (0.8994 ± 0.0133)*x* + (207.5 ± 65.6) for regions below and above 2500 cm^–1^, respectively; *y* represents the scaled vibrational wavenumber and *x* is the calculated harmonic vibrational wavenumber. These scaling equations were derived by fitting experimental vibrational wavenumbers of uracil to their corresponding theoretical values within the respective spectral ranges, as shown in Figure S1. Experimental vibrational wavenumbers recorded in the gas phase,^57^ solid N_2_, and solid Ar matrices^58^ are compared with the scaled harmonic and anharmonic vibrational wavenumbers computed with the B3LYP/aug-cc-pVTZ method in Table S1. A comparison of the experimental infrared spectrum of uracil in solid *p*-H_2_ with stick spectra based on scaled harmonic and anharmonic vibrational calculations is shown in Figure S2. This dual-scaling approach reduced the average absolute deviation between experiments and scaled harmonic vibrational wavenumbers to 8 ± 5 cm^–1^, which is smaller than the corresponding deviation of 14 ± 12 cm^–1^ for anharmonic vibrational wavenumbers. The average absolute deviation between the gas-phase experiment and *p-*H_2_ experiment is 9 ± 6 cm^–1^, while that between gas-phase experiments and scaled harmonic vibrational wavenumbers is 8 ± 7 cm^–1^. Electronic excitation energies and oscillator strengths were obtained using time-dependent density-functional theory (TD-DFT) at the B3LYP/aug-cc-pVTZ level of theory.

## 4 Results and Discussion

### 4.1 Potential-Energy Scheme of Hydrogen Reactions with Uracil

Detailed structural parameters for uracil (C_4_H_4_N_2_O_2_), calculated with the B3LYP/aug-cc-pVTZ method, are shown in Figure S3a. Possible products of the reaction H + uracil were explored using the CCSD­(T)/aug-cc-pVTZ//B3LYP/aug-cc-pVTZ method. The potential-energy schematics (PES) for the H-abstraction (left) and H-addition (right) channels are depicted in Figure 2. Energy values listed in black were computed with the CCSD­(T) method, while those in parentheses (olive) represent B3LYP/aug-cc-pVTZ results. According to CCSD­(T) calculations, H addition at the C5 position to form 5*H*-C_4_H_5_N_2_O_2_ **(1)** radical is the most exothermic channel (122 kJ mol^–1^) and proceeds via the smallest barrier of 15 kJ mol^–1^. The prefix 5*H* indicates the position of the added hydrogen. The H addition at the C6 position, yielding the 6*H*-C_4_H_5_N_2_O_2_ **(2)** radical, is the second-most exothermic (110 kJ mol^–1^) with a slightly larger barrier of 22 kJ mol^–1^. The H addition to the exocyclic oxygen at the C4 position, forming the 4*OH*-C_4_H_5_N_2_O_2_ **(3)** radical, is exothermic by 83 kJ mol^–1^ with a barrier of 39 kJ mol^–1^. The prefix 4*OH* indicates that the hydrogen added to the carbonyl moiety to form OH is on position C4. Other H-addition channels exhibit significantly higher barriers (>55 kJ mol^–1^) and are endothermic (except for the exothermic formation of 2O*H*-C_4_H_5_N_2_O_2_), rendering these pathways unlikely under the current experimental conditions at 3.2 K.

**Figure 2:**
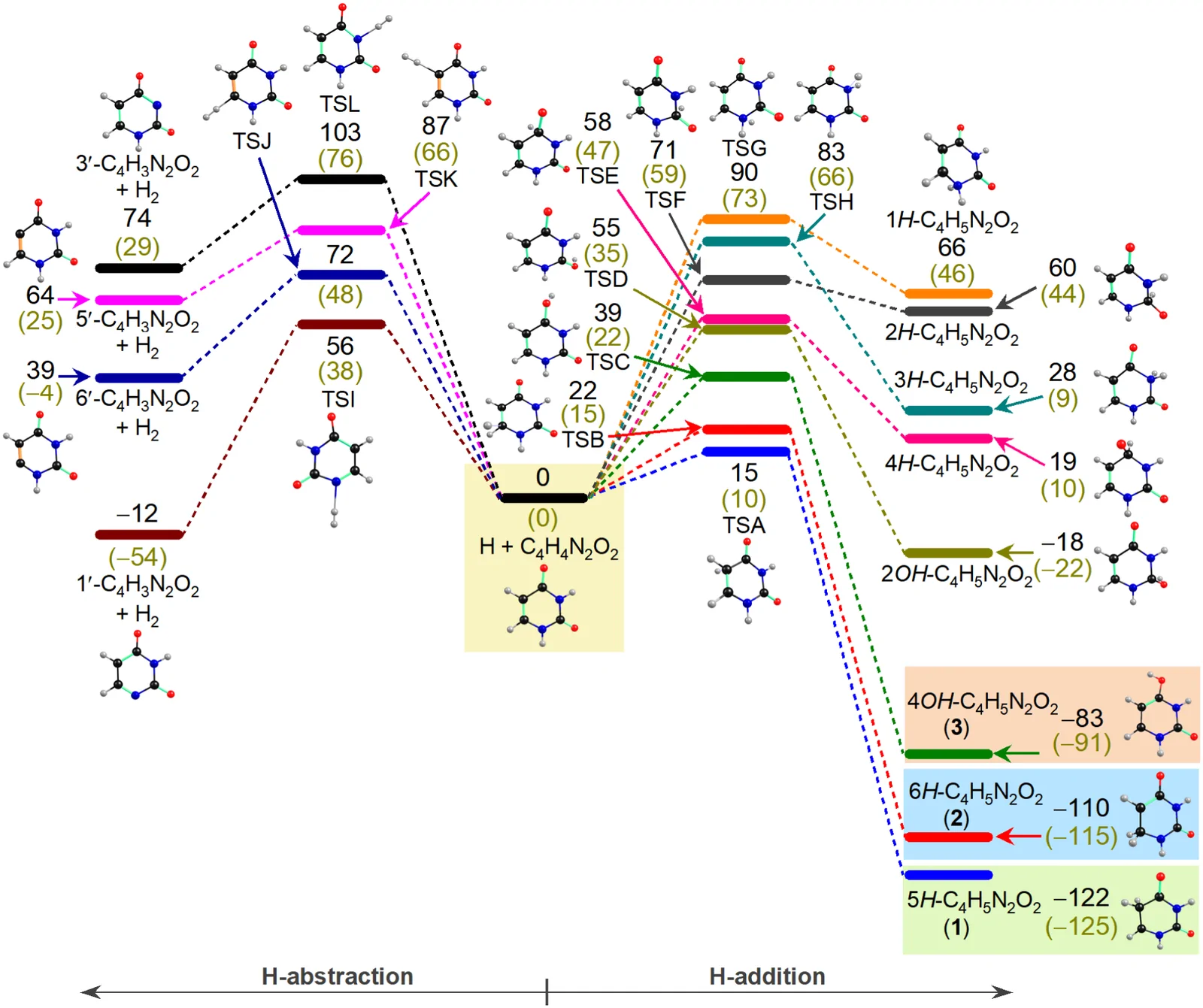
Potential-energy scheme of possible channels predicted for the reaction H + uracil (C_4_H_4_N_2_O_2_). H abstraction is shown on the left, and H addition is shown on the right. Energies (in kJ mol^–1^) relative to H + uracil were calculated using the CCSD­(T)/aug-cc-pVTZ//B3LYP/aug-cc-pVTZ method (black); values calculated using the B3LYP/aug-cc-pVTZ method are listed in parentheses (olive). Zero-point vibrational energies (ZPVE) from the B3LYP/aug-cc-pVTZ method were corrected.

Regarding H-abstraction channels, abstraction at the N1 site to form the 1′-C_4_H_3_N_2_O_2_ radical, in which the number with a prime indicates the H-abstraction site, is exothermic by only 12 kJ mol^–1^ but is hindered by a large barrier of 56 kJ mol^–1^. H-abstraction channels on the N3, C5, and C6 sites, producing the 3′-C_4_H_3_N_2_O_2_, 5′-C_4_H_3_N_2_O_2_, and 6′-C_4_H_3_N_2_O_2_ radicals, are endothermic by 74, 64, and 39 kJ mol^–1^, respectively, with barriers exceeding 72 kJ mol^–1^. Consequently, these abstraction pathways are considered unlikely to proceed under our experimental conditions. The optimized geometries of all possible H-addition and H-abstraction products are depicted in Figures S3–S5; those of the transition states involved in H-addition and H-abstraction reactions are presented in Figures S6 and S7, respectively. The optimized geometry of the end product 5,6-dihydrouracil (DHU, 5,6*H*-C_4_H_6_N_2_O_2_) after secondary H-addition is illustrated in Figure S8.

### 4.2 Identification of Features in Groups A, B, and C

Figure 3a depicts the IR spectrum in the region 1130–1800 cm^–1^ following the deposition of a uracil/Cl_2_/*p*-H_2_ (∼1/20/10000) matrix at 3.2 K for 6 h; the spectrum covering other spectral regions is presented in Figure S9a. The vibrational wavenumbers of uracil in solid *p*-H_2_ closely match those previously reported in solid N_2_ and solid Ar. Multiple bands observed in the region 1700–1800 cm^–1^ are consistent with the previously reported combinational bands, as detailed in Table S1.

**Figure 3:**
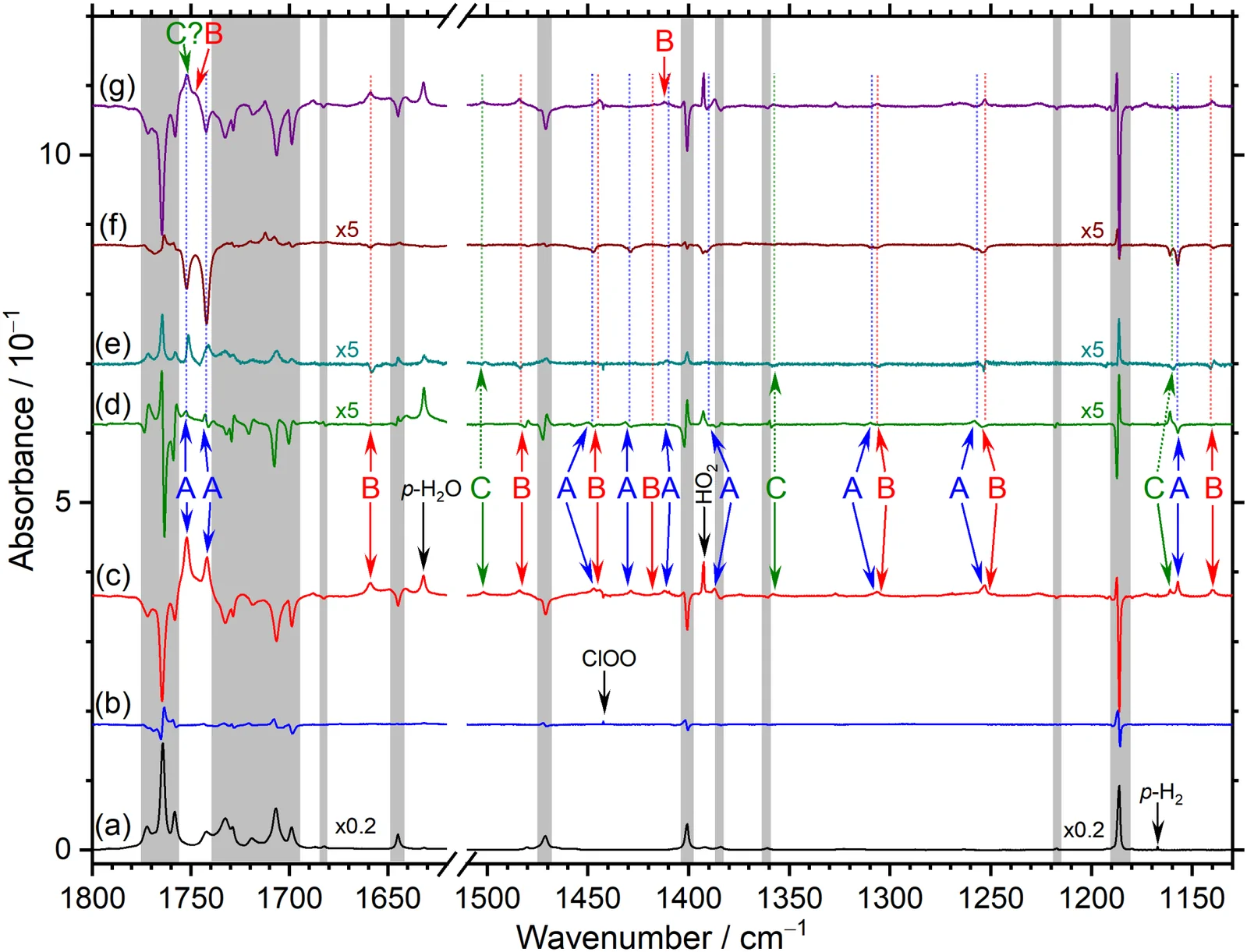
Representative spectra of a C_4_H_4_N_2_O_2_/Cl_2_/*p*-H_2_ (1/20/10000) matrix recorded at various stages of the experiment. (a) Spectrum recorded after deposition at 3.2 K for 6 h. (b) Difference spectrum after photolysis at 365 nm for 30 min. (c) Difference spectrum after additional full IR irradiation for 1 h. (d) Difference spectrum after maintaining the matrix in darkness for 10 h. (e) Difference spectrum after secondary irradiation at 460 nm for 20 min. (f) Difference spectrum after further irradiation at 399 nm for 20 min. (g) Processed spectrum of (c) + 0.57 × (f); the scaling factor was determined by diminishing the feature near 1157.2 cm^–1^ in (c). Bands in groups A–C are indicated with blue, red, and green arrows and labels, respectively. Areas shaded in gray indicate spectral regions that are interfered with by uracil absorption.

The matrix was irradiated with UV light at 365 nm for 30 min to generate Cl atoms. The resulting difference spectra are shown in Figures 3b and S9b. In this work, the difference spectrum was obtained by subtracting the spectrum recorded before a specific experimental step from that recorded afterward. Apart from the appearance of several first-derivative-shaped bands of parent, likely arising from a small shift of bands of uracil, the spectrum showed no significant change except a weak ClOO feature at 1442.1 cm^–1^,^59^ produced from the reaction of Cl with trace O_2_ impurities, together with the spin–orbit transition of Cl at 948.8 cm^–1^,^60^ confirming that the produced Cl atoms do not react significantly with uracil under these conditions. Subsequently, IR irradiation was employed for 60 min to generate H atoms via the reaction Cl + H_2_ (*v* = 1) → HCl + H. The difference spectrum (Figures 3c and S9c) shows new features for HCl (2894.1 cm^–1^), HCl clusters (2880–2865 cm^–1^), and HO_2_ (1392.4 and 1101.1 cm^–1^),^61^ indicating the formation of H atoms in the matrix. The amount of HO_2_ is small, hence its subsequent reactions with uracil are expected to be negligible. Additionally, the appearance of new features and the diminished bands of uracil confirm that H atoms reacted with uracil. No significant spectral changes were observed upon UV and IR irradiations in a control experiment performed without Cl_2_, as presented in Figure S10. Following IR irradiation, the matrix was maintained in darkness for 10 h to allow for H atom tunneling reactions; the difference spectrum is shown in Figures 3d and S9d. Subsequently, the matrix was exposed to secondary UV irradiation at 460 and 399 nm, each for 20 min, to categorize newly observed bands (Figures 3e,f and S9e,f). To resolve spectral congestion around 1750 and 1400 cm^–1^, the spectrum recorded after IR irradiation (Figures 3c and S9c) was processed by adding the spectrum (scaling factor 0.57, which was determined by diminishing the feature near 1157.2 cm^–1^ in Figure 3c) obtained upon 399 nm irradiation (Figures 3f and S9f) and displayed in Figures 3g and S9g; in this spectrum, bands in group A are expected to disappear and those in group B are positive.

Based on the photobehavior upon secondary irradiation, the new features were categorized into three groups. The intensities of bands in group A, assigned to 5*H*-C_4_H_5_N_2_O_2_ (1), increased by ∼6% after being maintained in darkness, remained nearly the same after irradiation at 460 nm, and decreased significantly by ∼69% after irradiation at 399 nm. The intensities of features in group B, assigned to 6*H*-C_4_H_5_N_2_O_2_ (2), decreased by ∼3%, ∼11%, and ∼4% after being maintained in darkness and irradiations at 460 and 399 nm, respectively. Lastly, the intensities of features in group C, assigned to 4*OH*-C_4_H_5_N_2_O_2_ (3), remained nearly unchanged after being maintained in darkness, decreased by ∼5% and ∼2% upon UV irradiation at 460 and 399 nm, respectively. The estimated mixing ratios and percentage variations for uracil and the three newly observed species are summarized in Table S2.

#### 4.2.1 Assignments of Bands in Groups A and B to 5*H*-C_4_H_5_N_2_O_2_ (1) and 6*H*-C_4_H_5_N_2_O_2_ (2) Radicals

Figures 4a and S11a, reproduced from Figures 3c,e,f,g and S9c,e,f,g, compare the observed features in group A (blue arrows and labels) and B (red arrows and labels) with the predicted IR stick spectra of the three most probable products from H addition to uracil, based on the scaled harmonic vibrational wavenumbers and IR intensities predicted with the B3LYP/aug-cc-pVTZ method, as depicted in Figures 4b–d and S11b–d.

**Figure 4:**
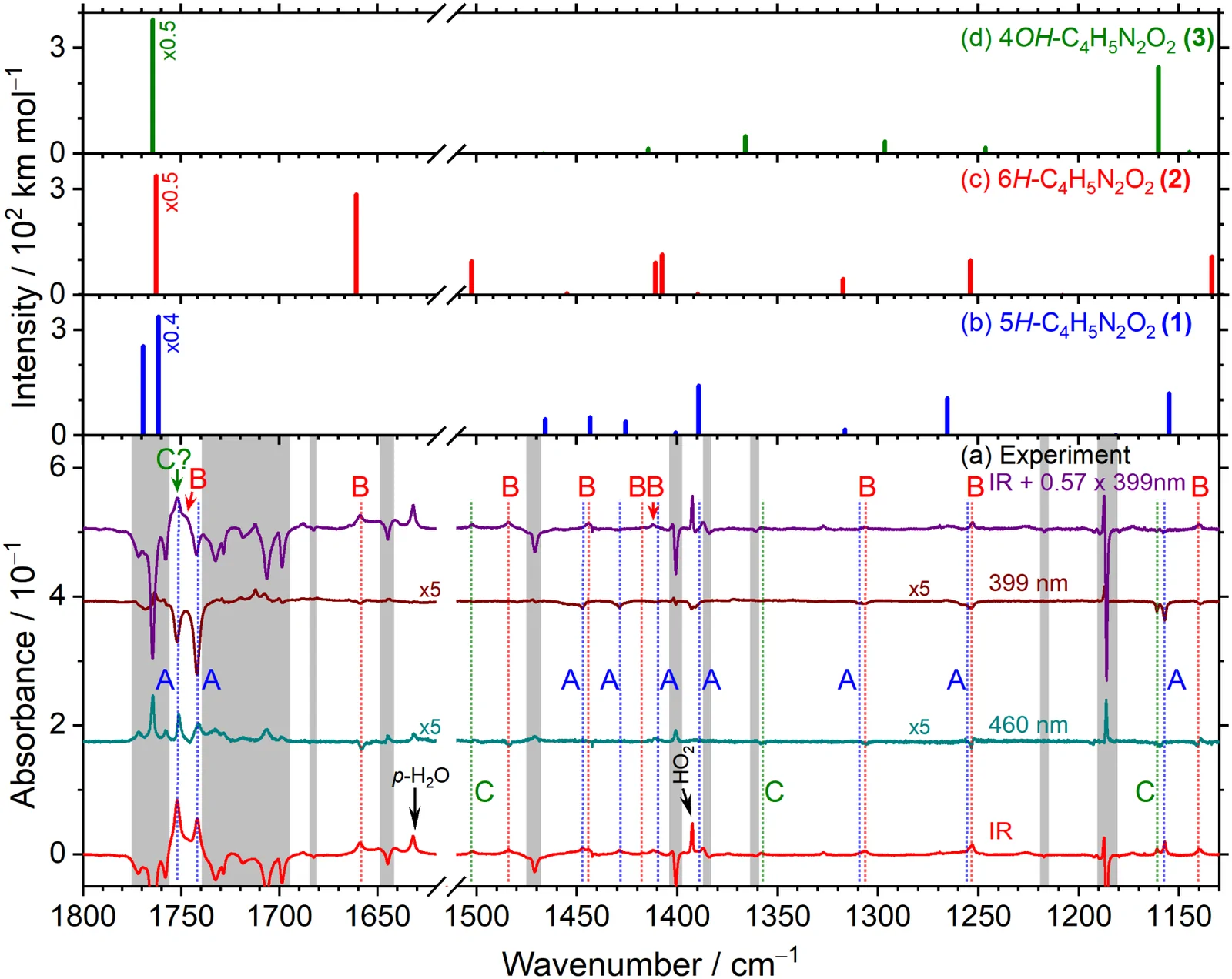
Comparison of bands in groups A–C with predicted IR stick spectra of possible products of H + uracil in the region 1130–1800 cm^–1^. (a) Experimental spectra: red, cyan, brown, and purple traces are taken from Figure 3c, e, f, and g, respectively. Bands in groups A–C are indicated with blue, red, and green arrows and labels, respectively. (b)–(d) Simulated IR stick spectra of possible candidates: (b) 5*H*-C_4_H_5_N_2_O_2_ **(1)**, (c) 6*H*-C_4_H_5_N_2_O_2_ **(2)**, and (d) 4*OH*-C_4_H_5_N_2_O_2_ **(3)**, according to scaled harmonic vibrational wavenumbers and IR intensities calculated with the B3LYP/aug-cc-pVTZ method. The areas shaded in gray indicate spectral regions that are interfered with by uracil absorption.

Lines in group A agree satisfactorily with the predicted spectrum of 5*H*-C_4_H_5_N_2_O_2_ **(1)** radical, shown in Figures 4b and S11b, in terms of vibrational wavenumbers and IR intensities. The observed wavenumbers and relative IR intensities are compared with scaled vibrational wavenumbers and predicted IR intensities of 5*H*-C_4_H_5_N_2_O_2_ **(1)** in Table 1. The primary structural change upon H addition at the C5 site is the conversion of the sp^2^-hybridized C5H group into a sp^3^-hybridized C5H_2_ moiety. This transformation introduces characteristic C5H_2_ vibrational modes, including two stretches (ν_4_ and ν_5_), scissor (ν_9_), wag (ν_13_), and twist (ν_15_), as predicted near 2980 (weak), 2827, 1443, 1316, and 1182 (weak) cm^–1^, respectively. Modes ν_5_, ν_9_, and ν_13_ were observed at 2832.3, 1447.2, and 1305.9 cm^–1^, respectively, whereas modes ν_4_ and ν_15_ were not observed because their predicted intensities were less than 2 km mol^–1^. The loss of aromaticity leads to significant elongation of the C5–C6 and C4–C5 bonds, from 1.343 Å to 1.485 Å and 1.456 Å to 1.518 Å, respectively (Figure S3b). However, the associated vibrational modes are heavily mixed. Furthermore, because the unpaired electron is localized on C6, adjacent to the nitrogen atom and the rest of the π-system, the tetrahedral “kink” at C5 makes the overall ring skeleton nonplanar.

**Table 1:** Comparison of Observed Vibrational Wavenumbers and Relative IR Intensities of Bands in Group a with Scaled Harmonic Vibrational Wavenumbers and IR Intensities of 5*H*-C_4_H_5_N_2_O_2_ **(1)** Predicted with the B3LYP/aug-cc-pVTZ Method

	Group A, 5*H*-C_4_H_5_N_2_O_2_ **(1)**				
	*p*-H_2_		B3LYP/aug-cc-pVTZ		
Mode^a^	ν/cm^–1^	Int.^b^/%	ν^c^/cm^–1^	Intensity/km mol^–1^	Mode description^d^
ν_1_	3462.2	7	3462	72	ν N1H
ν_2_	3436.7	^e^	3435	66	ν N3H
ν_5_	2832.3	3	2827	24	ν_a_ C5H_2_
ν_6_	1752.1	39	1769	253	ν C4=O/ν C2=O (*iph*)
ν_7_	1742.2	100	1762	844	ν C2=O/ν C4=O (*ooph*)
ν_8_	^e^		1466	46	ν_a_ C6N1C2
ν_9_	1447.2	7	1443	51	*sci* C5H_2_
ν_10_	1428.5	4	1426	39	*sci* C5H_2_/ν_a_ C5C6N1
ν_11_	1409.6	2	1401	8	δ N3H (*ip*)/ν N3C4
ν_12_	1390.6	12	1389	141	ν_a_ N1C2N3/ν_s_ C4C5C6
ν_13_	1305.9	2	1316	16	ω C5H_2_
ν_14_	1254.5	9	1265	105	ring *def*
ν_16_	1157.2	10	1155	119	δ C6H (*ip*)/δ N1H (*ip*)
ν_18_	^e^		951	11	γ C5H_2_
ν_21_	734.3	5	732	57	ring *def* (*oop*)
ν_22_	^e^		717	10	ring *breathing*
ν_23_	703.8	8	681	74	δ N3H (*oop*)

a Only modes with IR intensity >10 km mol^–1^ or those observed are listed.

b Integrated IR intensities as relative to the most intense band at 1742.2 cm^–1^ for 5*H*-C_4_H_5_N_2_O_2_ **(1)**.

c Harmonic vibrational wavenumbers scaled according to linear equations *y* = 0.9986*x* – 10.8 and *y* = 0.8994*x* + 207.5 for regions below and above 2500 cm^–1^, respectively; *x* is the calculated harmonic vibrational wavenumber.

d Approximate mode description; ν: stretch, δ: bend, ω: wag, γ: rock, *sci*: scissor, *def*: deformation, *ip*: in-plane, *oop*: out-of-plane, *iph*: in-phase, *ooph*: out-of-phase, s: symmetric, a: antisymmetric.

e Interference due to the absorption of uracil.

The two most intense bands associated with the C4=O and C2=O stretching (ν_6_ and ν_7_) modes were predicted near 1769 and 1762 cm^–1^ and observed at 1752.1 and 1742.2 cm^–1^, close to the corresponding features observed at 1764.4 and 1733.6 cm^–1^ for uracil, indicating that the C=O bonds were not altered much by the hydrogenation, consistent with a structure with a radical center isolated from the two C=O bonds. The calculations indicate that the two C=O stretches in **(1)** are coupled, with ν_6_ and ν_7_ corresponding to in-phase and out-of-phase motions, whereas those of uracil are nearly uncoupled, with C2=O ∼30 cm^–1^ higher than C4=O.

The two NH stretching modes (ν_1_ and ν_2_) were predicted near 3462 and 3435 cm^–1^ and observed at 3462.2 and 3436.7 cm^–1^, respectively. The ν_1_ mode (N1H stretch) appears at a slightly smaller wavenumber than that of uracil (3479.0 cm^–1^), consistent with a small increase in the N–H bond length from 1.006 to 1.007 Å, attributable to the neighboring radical center. In contrast, the ν_2_ mode (N3H stretch) shows a wavenumber (3436.7 cm^–1^) close to that of uracil (3431.3 cm^–1^) because this part of the structure was nearly unaffected by H addition at the C5 site. The ring deformation modes were predicted near 1389 cm^–1^ (ν_12_) and 1265 cm^–1^ (ν_14_), and observed at 1390.6 and 1254.5 cm^–1^, respectively. Other modes predicted near 1426 (ν_10_, C5H_2_ scissor coupled with antisymmetric C5C6N1 stretch), 1401 (ν_11_, in-plane N3H bend and N3C4 stretch), 1155 (ν_16_, in-plane C6H and N1H bends), 732 (ν_21_, out-of-plane ring deformation), and 681 (ν_23_, out-of-plane N3H bend) cm^–1^ were observed at 1428.5, 1409.6, 1157.2, 734.3, and 703.8 cm^–1^, respectively. Most predicted bands of radical **(1)** in our detection range with IR intensity greater than 10 km mol^–1^ were observed, except bands predicted near 1466, 951, and 717 cm^–1^ (with IR intensities 46, 11, and 10 km mol^–1^), corresponding to ν_8_, ν_18_, and ν_22_ modes, could not be definitively identified due to the severe interference by the parent absorption. In total, 14 bands in group A are unambiguously assigned to the 5*H*-C_4_H_5_N_2_O_2_ **(1)** radical, with an average absolute deviation from scaled harmonic vibrational wavenumbers of only 8.1 ± 6.9 cm^–1^; the deviation from anharmonic vibrational wavenumbers is 17.2 ± 15.0 cm^–1^.

The vibrational wavenumbers and IR intensities of bands in group B, shown as red arrows and labels in Figures 4a and S11a, agree satisfactorily with those of the predicted spectrum of the 6*H*-C_4_H_5_N_2_O_2_ **(2)** radical, depicted in Figures 4c and S11c. The observed and predicted vibrational wavenumbers and IR intensities of radical **(2)** are summarized in Table 2. Similar to radical **(1)**, the primary geometrical change from uracil to radical **(2)** involves the conversion of the sp^2^-hybridized C6H moiety to a sp^3^-hybridized C6H_2_ moiety, leading to the elongation of bond lengths of the C5–C6 and C6–N1, from 1.343 Å to 1.479 Å and 1.371 Å to 1.450 Å, respectively (Figure S3c). However, unlike the case of radical **(1)**, because C5 is part of a conjugated system involving the C4=O carbonyl group, the ring skeleton of radical **(2)** remains planar.

**Table 2:** Comparison of Observed Vibrational Wavenumbers and Relative IR Intensities of Bands in Group B with Scaled Harmonic Vibrational Wavenumbers and IR Intensities of 6*H*-C_4_H_5_N_2_O_2_ **(2)** Predicted with the B3LYP/aug-cc-pVTZ Method

		Group B, 6*H*-C_4_H_5_N_2_O_2_ **(2)**				
		*p*-H_2_		B3LYP/aug-cc-pVTZ		
Mode^a^	Sym.	ν/cm^–1^	Int./%^b^	ν^c^/cm^–1^	Int./km mol^–1^	Mode description^d^
ν_1_	a′	3471.2	26	3478	56	ν N1H
ν_2_	a′	3450.2	32	3450	74	ν N3H
ν_4_	a′	^e^		2855	35	ν_s_ C6H_2_
ν_5_	a′	1747.4	^e^	1763	676	ν C2=O
ν_6_	a′	1659.2	100	1661	285	ν C4=O
ν_7_	a′	1484.2	39	1502	96	*sci* C6H_2_/ν N1C2
ν_8_	a′	1444.6	3	1455	5	δ N1H (*ip*)/*sci* C6H_2_
ν_9_	a′	1417.5	27	1411	92	ν_a_ N1C2N3
ν_11_	a′	1412.3	^f^	1407	115	ω C6H_2_/ν C4C5
ν_12_	a′	1304.9	25	1317	46	ν_a_ C5C6N1/δ C5H (*ip*)
ν_13_	a′	1253.4	41	1254	98	ν N1C6/ν N3C4 (*ooph*)
ν_14_	a′	1139.5	39	1134	110	ν N1C6/ν C2N3 (*ooph*)
ν_20_	a″	^e^		2853	12	ν_a_ C6H_2_
ν_23_	a″	^e^		928	11	ring *def*
ν_24_	a″	747.2	13	744	32	ring *def* (*oop*)
ν_25_	a″	704.2	^f^	709	51	ring *def* (*oop*)
ν_26_	a″	608.7	29	614	52	δ N3H (*oop*)

a Only modes with IR intensity >10 km mol^–1^ or those observed are listed.

b Integrated IR intensities as relative to the most intense band at 1659.2 cm^–1^ for 6*H*-C_4_H_5_N_2_O_2_ **(2)**.

c Harmonic vibrational wavenumbers scaled according to linear equations *y* = 0.9986 *x* – 10.8 and *y* = 0.8994 *x* + 207.5 for regions below and above 2500 cm^–1^, respectively; *x* is the calculated harmonic vibrational wavenumber.

d Approximate mode description; ν: stretch, δ: bend, ω: wag, *sci*: scissor, *def*: deformation, *ip*: in-plane, *oop*: out-of-plane, *ooph*: out-of-phase, s: symmetric, a: antisymmetric.

e Interference due to the absorption of uracil.

f Interference due to the absorption of 5*H*-C_4_H_5_N_2_O_2_ **(1)**.

Accordingly, IR vibrational modes involving the C6H_2_ group, including symmetric and *anti*-symmetric stretches (ν_4_ and ν_20_), scissor (ν_7_), wag (ν_11_), twist (ν_21_), and rock (ν_22_), were predicted near 2855, 2853, 1502, 1407, 1208 (weak), and 929 (weak) cm^–1^. However, only the scissor (ν_7_) and wag (ν_11_) were observed at 1484.2 and 1412.3 cm^–1^; ν_21_ and ν_22_ were predicted to have intensities less than 3 km mol^–1^, and symmetric and *anti*-symmetric stretches were interfered with by absorption of uracil. Although the vibrational mode associated with C6H_2_ wag coupled with C4C5 stretch (ν_11_) could not be clearly identified in the spectrum recorded after IR irradiation (bottom trace of Figure 4a) due to interference from radical **(1)**, it was clearly observed at 1412.3 cm^–1^ in the processed spectrum, depicted in the top trace of Figure 4a.

The band corresponding to the C4=O stretching (ν_6_) mode, predicted near 1661 cm^–1^ and observed at 1659.2 cm^–1^, is markedly red-shifted from the corresponding value of 1733.6 cm^–1^ of uracil due to the delocalization with the radical center on the adjacent C5 atom, as reflected by an increase in the bond length of C4=O from 1.214 to 1.223 Å. Although the C2=O stretching (ν_5_) mode, predicted near 1763 cm^–1^, could not be clearly identified due to the severe interference from radical **(1)**, it was clearly visible at 1747.4 cm^–1^ as a shoulder to the 1751.7 cm^–1^ band in the processed spectrum (top trace of Figure 4a). Moreover, the N3H stretch (ν_2_) was predicted near 3450 cm^–1^ and observed at 3450.2 cm^–1^, whereas the N1H stretch (ν_1_) predicted near 3478 cm^–1^ is assigned to a band at 3471.2 cm^–1^. Additionally, bands at 1444.6 (ν_8_, in-plane N1H bend coupled with C6H_2_ scissor), 1417.5 (ν_9_, antisymmetric N1C2N3 stretch), 1304.9 (ν_12_, *anti*-symmetric C5C6N1 stretch coupled with in-plane C5H bend), 1253.4 (ν_13_, out-of-phase N1C6 and N3C4 stretches), 1139.5 (ν_14_, out-of-phase N1C6 and C2N3 stretches), 747.2 (ν_24_, out-of-plane ring deformation), 704.2 (ν_25_, out-of-plane ring deformation), and 608.7 (ν_26_, out-of-plane N3H bend) cm^–1^, predicted near 1455, 1411, 1317, 1254, 1134, 744, 709, and 614 cm^–1^, were identified. A band predicted near 928 (ν_23_) could not be identified due to interference from uracil.

Within our detection range, out of the 17 bands of 6*H*-C_4_H_5_N_2_O_2_ **(2)** radical with predicted IR intensities >10 km mol^–1^, 14 bands were observed. We hence unambiguously assign features in group B to the 6*H*-C_4_H_5_N_2_O_2_ **(2)** radical. The average absolute deviation from scaled harmonic vibrational wavenumbers of **(2)** is 6.7 ± 5.3 cm^–1^, while that from anharmonic vibrational wavenumbers is 14.5 ± 9.8 cm^–1^.

#### 4.2.2 Tentative Assignments of Bands in Group C to the 4*OH*-C_4_H_5_N_2_O_2_ Radical

The observed bands in group C, denoted by green arrows and labels in Figures 4a and S11a, agree satisfactorily with the IR stick spectrum of 4*OH*-C_4_H_5_N_2_O_2_ **(3)** radical, shown in Figures 4d and S11d. Table 3 compares the experimental values for bands in group C with the predicted scaled harmonic vibrational wavenumbers and IR intensities of 4*OH*-C_4_H_5_N_2_O_2_ **(3)**. The primary geometrical transformation from uracil to radical **(3)** is the conversion of the C4=O moiety into the C4–OH group upon H addition, resulting in a significant elongation of the C–O bond from 1.214 to 1.361 Å, shifting the C4O stretching mode to ∼1246 cm^–1^ (ν_13_). Unfortunately, this mode could not be identified, partly due to its small intensity (17 km mol^–1^) and partly due to possible interference from absorption bands of **(1)** and **(2)** near 1254 cm^–1^. The most definitive evidence for the formation of radical **(3)** is hence the observation of the OH stretching mode (ν_1_) at 3651.7 cm^–1^ (predicted near 3651 cm^–1^). In addition to this characteristic band, three additional bands were unambiguously assigned. The C5=C6 stretch coupled with the N3C4 stretch (ν_8_) was predicted near 1510 cm^–1^ and observed at 1502.4 cm^–1^. The C5=C6 stretch coupled with a C2N3 stretch (ν_11_) was predicted near 1366 cm^–1^ and observed at 1358.6 cm^–1^. The most intense mode observed at 1161.1 cm^–1^ corresponds to the in-plane C4OH bending vibration (ν_14_), which was predicted near 1160 cm^–1^. A band at 1751.7 cm^–1^ in the processed spectrum (top trace of Figure 4a) is tentatively assigned to the C2=O (ν_6_) stretch, predicted near 1765 cm^–1^. Because of the small mixing ratio, only bands with predicted IR intensities greater than 50 km mol^–1^ were observed.

**Table 3:** Comparison of Observed Vibrational Wavenumbers and Relative IR Intensities of Bands in Group C with Scaled Harmonic Vibrational Wavenumbers and IR Intensities 4*OH*-C_4_H_5_N_2_O_2_ **(3)** Predicted with the B3LYP/aug-cc-pVTZ Method

		Group C, 4*OH*-C_4_H_5_N_2_O_2_ **(3)**				
		*p*-H_2_		B3LYP/aug-cc-pVTZ		
Mode^a^	Sym.	ν/cm^–1^	Int.^b^/%	ν^c^/cm^–1^	Int./km mol^–1^	Mode description^d^
ν_1_	a′	3651.7	51	3651	89	ν O4H
ν_2_	a′	^e^		3486	49	ν N1H
ν_3_	a′	^e^		3481	109	ν N3H
ν_6_	a′	1751.7(?)	^e^	1765	761	ν C2=O
ν_7_	a′	^e^		1600	134	ν C4=C5
ν_8_	a′	1502.4	57	1510	97	ν C5=C6/ν N3C4 (*ooph*)
ν_10_	a′	^e^		1414	15	ν C6N1/ν N3C4 (*ooph*)
ν_11_	a′	1358.6	27	1366	50	ν C5=C6/ν C2N3 (*ooph*)
ν_12_	a′	^f^,^g^		1297	35	ν_a_ N1C2N3
ν_13_	a′	^f^,^g^		1246	17	ν C4O
ν_14_	a′	1161.1	100	1160	247	δ C4OH
ν_17_	a′	^e^		996	12	ring *def* (*ip*)
ν_18_	a′	^e^		937	50	ring *def* (*ip*)
ν_23_	a″	^e^		792	18	δ C5H (*oop*)
ν_24_	a″	^f^		734	43	ring *def* (*oop*)
ν_26_	a″	^e^		550	74	ring *def* (*oop*)

a Only modes with IR intensity >10 km mol^–1^ or those observed are listed.

b Integrated IR intensities as relative to the most intense band at 1502.4 cm^–1^ of 4*OH*-C_4_H_5_N_2_O_2_ **(3)**.

c Harmonic vibrational wavenumbers scaled according to linear equations *y* = 0.9728 *x* + 12.8 and *y* = 0.8767 *x* + 294.4 for regions below and above 2500 cm^–1^, respectively; *x* is the calculated harmonic vibrational wavenumber.

d Approximate mode description; ν: stretch, δ: bend, *def*: deformation, *ip*: in-plane, *oop*: out-of-plane, *ooph*: out-of-phase.

e Interference due to the absorption of uracil.

f Interference due to the absorption of 5*H*-C_4_H_5_N_2_O_2_ **(1)**.

g Interference due to the absorption of 6*H*-C_4_H_5_N_2_O_2_ **(2)**.

Five additional bands were predicted to have IR intensities ∼50 km mol^–1^ or greater, locating near 3486 (ν_2_, N1H stretch), 3481 (ν_3_, N3H stretch), 1600 (ν_7_, C4=C5 stretch), 937 (ν_18_, in-plane ring deformation), and 550 (ν_26_, out-of-plane ring deformation) cm^–1^, but they could not be identified because of the interference from the parent absorption. Moreover, weaker bands predicted at 1414 (ν_10_), 1297 (ν_12_), and 1246 (ν_13_) might be interfered with by bands of the parent or radical **(1)** or **(2)**. Because only five bands were identified, we tentatively assign these features in group C to the 4*OH*-C_4_H_5_N_2_O_2_ **(3)** radical. The average absolute deviation from scaled harmonic vibrational wavenumbers of **(3)** is 4.4 ± 2.3 cm^–1^, while that from anharmonic vibrational wavenumbers is 9.6 ± 6.5 cm^–1^.

The primary difference between 5*H*-C_4_H_5_N_2_O_2_ **(1)** and 6*H*-C_4_H_5_N_2_O_2_ **(2)** radicals is the site of hydrogen addition. In radical **(2)**, delocalization of the unpaired electron at the C5 position leads to elongation of the C4–O bond, resulting in a noticeable red shift of the corresponding C4 = O band from 1742.2 cm^–1^ in **(1)** to 1659.2 cm^–1^ in **(2).** This shift represents the most significant spectral difference between radicals **(1)** and **(2)**. For 4*OH*-C_4_H_5_N_2_O_2_ **(3)**, the OH stretching vibration at 3651.7 cm^–1^ and the intense C4OH bending vibration are the characteristic modes, which are absent in uracil and radicals **(1)** and **(2)**. Furthermore, we compared features in groups A–C with the simulated IR stick spectra of other product candidates predicted in H-addition channels, including 1*H*-C_4_H_5_N_2_O_2_, 2*H*-C_4_H_5_N_2_O_2_, 3*H*-C_4_H_5_N_2_O_2_, 4*H*-C_4_H_5_N_2_O_2_, and 2*OH*-C_4_H_5_N_2_O_2_, and H-abstraction channels, including 1′-C_4_H_3_N_2_O_2_, 3′-C_4_H_3_N_2_O_2_, 5′-C_4_H_3_N_2_O_2_, and 6′-C_4_H_3_N_2_O_2_, as depicted in Figures S12 and S13; these IR stick spectra do not agree with observed spectra in groups A–C.

#### 4.2.3 Tentative Assignments of Group D to 5,6*H*-C_4_H_6_N_2_O_2_ (DHU)

The end-product 5,6-dihydrouracil (DHU, 5,6*H*-C_4_H_6_N_2_O_2_) is expected to form upon secondary hydrogen addition to **(1)** or **(2)** in hydrogen-rich experiments. A comparison of the observed spectrum following IR Irradiation with the stick spectra of DHU based on those previously reported in Ar and N_2_ matrices,^62^ along with the predicted IR stick spectra using the B3LYP/aug-cc-pVTZ method, is depicted in Figure S14. Unfortunately, spectral congestion and interference from uracil, radicals **(1)** and **(2)** prevent an unambiguous assignment of DHU. To compensate for the loss of uracil after IR irradiation, we added the spectra of uracil recorded after deposition (Figures 3a and S9a) with a scaling factor of 0.21 to the difference spectra recorded after IR irradiation (Figures 3c and S9c), as presented in Figure S15a (top trace). Four weak features at 1758.6, 1362.1, 1190.9, and 670.3 cm^–1^, denoted by brown bands and labels as group D, which were absent in the H-deficient experiment (middle trace of Figure S15a), may be tentatively assigned to DHU, as their positions are consistent with the previously reported features of DHU isolated in solid N_2_ and Ar matrices^62^ and with the simulated IR stick spectra of DHU (Table S3).

### 4.3 Photolytic Behavior

The decay behavior of these radicals under secondary irradiation is supported by time-dependent (TD)-DFT calculations. Figure S16 shows the vertical-excitation UV stick spectra of uracil, 5*H*-C_4_H_5_N_2_O_2_ **(1)**, 6*H*-C_4_H_5_N_2_O_2_ **(2)**, and 4*OH*-C_4_H_5_N_2_O_2_ **(3)** computed with the TD-B3LYP/aug-cc-pVTZ method, along with contours convoluted using a full-width-half-maximum (fwhm) of 20 nm. The corresponding electronic transitions and oscillator strength of these species are summarized in Tables S4–S7. The frontier molecular orbitals and associated transitions of **(1)**, **(2)**, and **(3)** are illustrated in Figures S17a, S17b, and S18, respectively.

The decrease in intensity of bands in group A, assigned to radical **(1)**, upon irradiation at 399 nm aligns with the first electronic transition, HOMO­(α) → LUMO­(α), of **(1)** predicted near 402 nm with an oscillator strength of 0.0097. The decay of bands in groups B and C, assigned to radicals **(2)** and **(3)**, at 460 nm aligns with electronic excitations of **(2)** and **(3)** associated with HOMO–2­(β) → LUMO­(β) predicted near 420 and 465 nm, respectively. Moreover, bands in group B also decreased to some extent after irradiation at 399 nm, corresponding to a third electronic transition near 400 nm, with an oscillator strength of 0.0029. In contrast, no absorption is predicted for uracil at wavelengths greater than 266 nm. Crucially, all identified electronic transitions involve excitation to the LUMO, which has antibonding character relative to the C–H bonds in radicals **(1)** and **(2)** and the O–H bond in radical **(3)**. This character explains why UV irradiation facilitates the cleavage of the added H atom, effectively reforming the uracil parent.

### 4.4 Temporal Profiles and Reaction Mechanism

The temporal evolution of uracil and its hydrogenation products was tracked through two distinct types of experiments: an H-deficient regime with [H]_0_/[uracil]_0_ ≈ 2.1 and [uracil]_0_ = 47.2 ppm and an H-rich regime with [H]_0_/[uracil]_0_ ≈ 7.9 and [uracil]_0_ = 45.3 ppm. The results are shown in Figure 5, and the estimated mixing ratios after each step are summarized in Table S2. To minimize uncertainties in calculated IR intensities, mixing ratios were derived by averaging data across multiple spectral bands (see Supporting Information Note 1 and Table S8). In general, comparisons of mixing ratios between species should be used with caution, but percentage variations in a specific species’ mixing ratio across various stages of an experiment are considered reliable.

**Figure 5:**
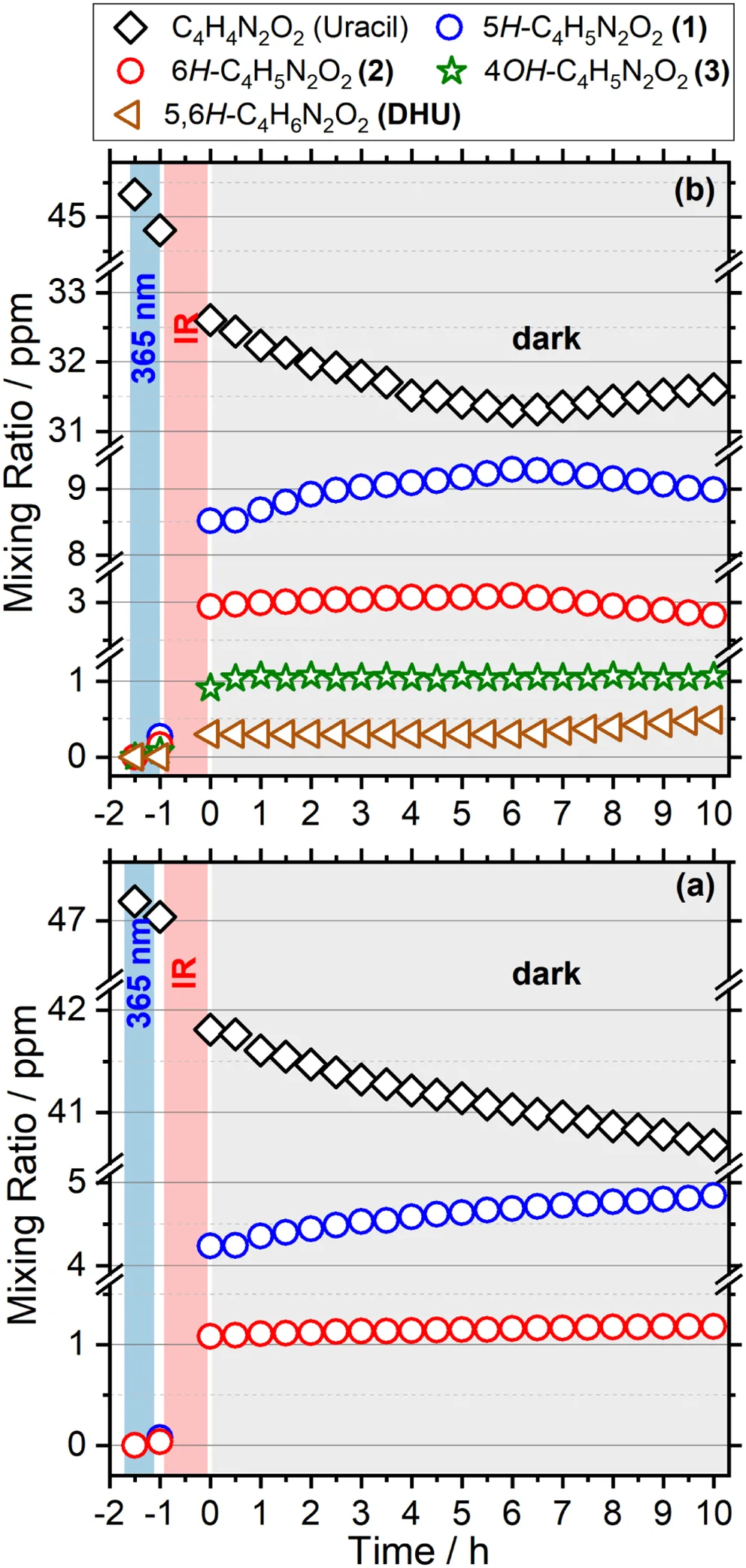
Temporal evolution of estimated mixing ratios of C_4_H_4_N_2_O_2_ (uracil) and several products produced in the reaction H + uracil. Periods of UV and IR irradiation are shaded in blue and red, respectively; the dark period is in gray. Time zero is set immediately after IR irradiation stops. (a) H-deficient experiment: [H]_0_/[uracil]_0_ ≈ 2.1; [uracil]_0_ = 47.2 ppm. (b) H-rich experiment: [H]_0_/[uracil]_0_ ≈ 7.9; [uracil]_0_ = 45.3 ppm. [H]_0_ was estimated from [HCl]_0_.

In the H-deficient experiment, the mixing ratio of uracil decreased by 5.2 ppm following IR irradiation, while that of radicals **(1)** and **(2)** increased by ∼4.1 and ∼1.1 ppm, respectively, as shown in Figure 5a and Table S2. During the 10-h dark period, a clear anticorrelation was observed: uracil continued to decrease by 1.1 ppm while radical **(1)** increased by ∼0.6 ppm and radical **(2)** increased slightly by ∼0.1 ppm. The reaction H + uracil proceeds more efficiently under IR irradiation than in the dark, which is attributed to the continuous generation of H atoms via the reaction of Cl with *p*-H_2_ during IR irradiation. In contrast, the observed reactivity in darkness is likely driven by the gradual chemical diffusion of residual H atoms within the matrix, followed by reactions with uracil. The observation of significantly more radical **(1)** than radical **(2)** agrees with the predicted barriers of 15 and 22 kJ mol^–1^, respectively. Despite these barriers, these reactions proceed in the dark at 3.2 K, providing strong evidence of H atom tunneling. The absence of radical **(3)** in this regime is consistent with its predicted significantly larger barrier (39 kJ mol^–1^) compared to those of **(1)** and **(2)**. This is also consistent with a previous study in the gas-phase by Wolken et al.,^28^ who reported enhanced stability of radical **(1)** over radical **(3)** at 298 K.

In the H-rich experiment (Figure 5b), the higher flux of H atoms enabled the detection of the minor product, radical **(3)**, whose formation requires a larger barrier. In this experiment, uracil decreased by ∼12.2 ppm after IR exposure, whereas those of the hydrogenated products **(1)**, **(2)**, and **(3)** increased by ∼8.2, ∼2.8, and ∼0.9 ppm, respectively. Interestingly, the temporal behavior in darkness differed after 6 h. Uracil decreased by ∼1.3 ppm during the first 6 h and subsequently increased by ∼0.3 ppm over the next 4 h, whereas radicals **(1)** and **(2)** increased by ∼0.8 and ∼0.1 ppm for the initial 6 h, and decreased by ∼0.3 and ∼0.2 ppm in the subsequent 4 h, respectively, showing the anticorrelation between uracil and **(1)/(2)**. The mixing ratio of **(3)** was too small to clearly demonstrate its anticorrelation with uracil. This reversal in mixing ratios over time suggests that at high H atom concentrations, secondary reactions become competitive. Furthermore, examination of the features of DHU reveals that they increased by ∼0.3 ppm after IR irradiation and by ∼0.2 ppm after 10 h in darkness, as depicted in Figure S19 and Table S2; these mixing ratios may be subject to large errors due to the weakly identified bands.

The potential energy scheme (PES) for secondary H reactions, illustrated in Figure S20, shows that H atoms can react further with radicals **(1)** and **(2)** via barrierless H-abstraction pathways to reform the parent uracil:

4 $${H+5H‐C}_{4}H_{5}N_{2}O_{2}\left(1\right)\to C_{4}H_{4}N_{2}O_{2}\left(\mathrm{uracil}\right){+H}_{2}$$

5 $${H+6H‐C}_{4}H_{5}N_{2}O_{2}\left(2\right)\to C_{4}H_{4}N_{2}O_{2}\left(\mathrm{uracil}\right){+H}_{2}$$

In addition, further hydrogen addition to radicals **(1)** or **(2)** leads to the formation of 5,6-dihydrouracil (DHU, 5,6*H*-C_4_H_6_N_2_O_2_) via barrierless H-addition:

6 $${H+5H‐C}_{4}H_{5}N_{2}O_{2}\left(1\right)\to {5,6H‐C}_{4}H_{6}N_{2}O_{2}\left(\mathrm{DHU}\right)$$

7 $${H+6H‐C}_{4}H_{5}N_{2}O_{2}\left(2\right)\to {5,6H‐C}_{4}H_{6}N_{2}O_{2}\left(\mathrm{DHU}\right)$$

The hydrogen abstraction of DHU by H atoms to reform **(1)** and **(2)** has a barrier of ∼35 kJ mol^–1^ and is considered to be less favored.

From Figure 5b, radicals **(1)** and **(2)**, rather than DHU, are the major products even in H-rich experiments. Furthermore, even though the reaction of uracil with H atoms may still play a significant role, uracil increased by ∼0.3 ppm, as compared with the smaller increase in DHU (approximately estimated to be 0.2 ppm) in the latter 4 h in darkness in reactions between H atoms and **(1)**/**(2)**. These experimental results indicate that reactions (4) and (5) are more important than reactions (6) and (7). In contrast, in the case of hydrogen reactions with formamide (H_2_NCHO), the stable end-product HNCO was the major product, while the radical H_2_NCO acted as a transient intermediate with a small mixing ratio,^33^ indicating that the reaction of H + H_2_NCO → HNCO + H_2_ is more important than H + H_2_NCO → H_2_NCHO. It is interesting to note that, in both cases, H-abstraction of the radical is more important than H-addition, even though H + uracil proceeds to form the radical via H-addition, whereas the H + formamide proceeds to form the radical via H-abstraction. Whether the H-abstraction channel is, in general, more important than the H-addition channel in the reaction of H + radicals requires further experimental and theoretical investigations.

The reaction channels derived from our observations are summarized in Figure 6. The reactions of H atoms with uracil in *p-*H_2_ at low temperature, either during UV/IR irradiation or in darkness, produced radical **(1)** via H-addition at site C5 as the major channel and radical **(2)** via addition at site C6 as the minor channel. Under H-rich conditions, the formation of radical **(3)**, via H-addition to the carbonyl oxygen at site C4, and DHU, via secondary H-addition to the radicals **(1)** and **(2**), was observed as a minor channel. The relative branching among radicals **(1)**, **(2)**, and **(3)** is consistent with the barriers predicted from quantum-chemical calculations. In H-rich experiments, the mixing ratios of uracil and **(1)** show a clear *anti*-correlation relationship, and the increase in mixing ratio in **(1)** is much larger than that of DHU, indicating that H-abstraction to regenerate uracil is more important than H-addition to form DHU for radicals **(1)** and **(2)**.

**Figure 6:**
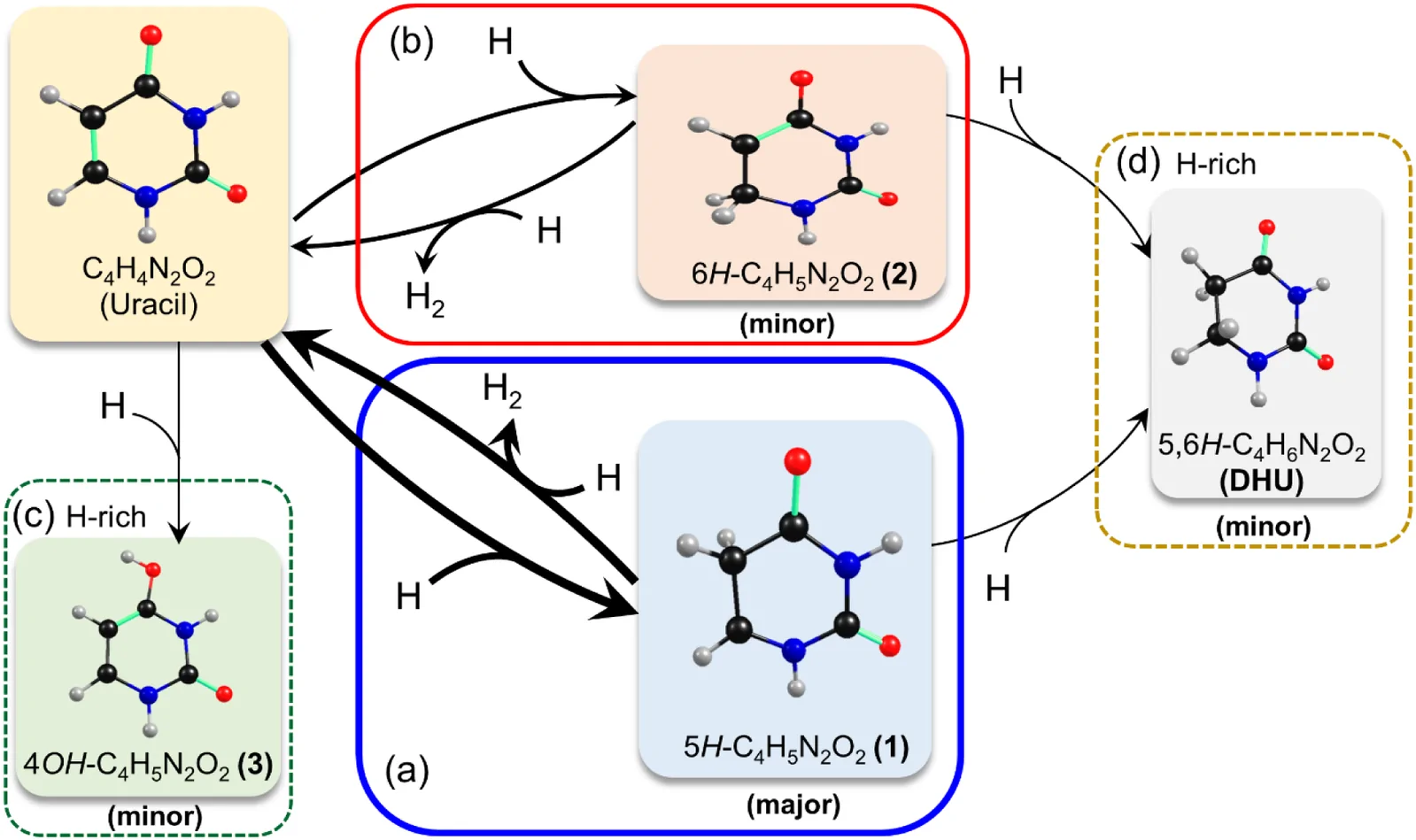
Proposed reaction mechanism for the reaction H + uracil. (a) The major channel: H-addition and H-abstraction reactions between uracil and 5*H*-C_4_H_5_N_2_O_2_ **(1)**. (b) The minor channel: H-addition and H-abstraction reactions between uracil and 6*H*-C_4_H_5_N_2_O_2_ **(2)**. (c) The minor channel: H-addition of uracil to form 4*OH*-C_4_H_5_N_2_O_2_ **(3)**. (d) The minor channel: 5*H*-C_4_H_5_N_2_O_2_ **(1)** and 6*H*-C_4_H_5_N_2_O_2_ **(2)** further produce 5,6*H*-C_4_H_6_N_2_O_2_ (DHU) via a second H addition. Both **(3)** and DHU were observed only in the H-rich experiment. Solid lines represent the observed pathways, while dotted lines indicate minor pathways involving DHU. The relative line thickness of arrows indicates relative importance.

### 4.5 Astronomical Implications

Our experiments demonstrated that the reaction of uracil with hydrogen atoms leads to three distinct monohydrogenated radicals: 5*H*-C_4_H_5_N_2_O_2_ **(1)**, 6*H*-C_4_H_5_N_2_O_2_ **(2)**, and 4*OH*-C_4_H_5_N_2_O_2_ **(3)**, corresponding to H addition to the C5, C6, and O4 sites, respectively, and one dihydrogenated product: 5,6*H*-C_4_H_6_N_2_O_2_ (DHU); assignments on the latter two are tentative. These observations provide direct experimental evidence that multiple H-addition pathways are accessible under the low-temperature, dark conditions characteristic of dense molecular clouds. However, the H-addition channels leading to the formation of **(3)** and DHU were observed only in H-rich experiments. Alternative pathways involving hydrogen abstraction or addition at other sites are hindered by large barriers or endothermicity, rendering them nonviable in cold interstellar environments.

These singly hydrogenated radicals may serve as key intermediates in the interstellar formation of nucleobases. For example, in the computational studies of the gas-phase formation of interstellar nucleobases from dehydrogenated formamide and vinyl cyanide, Lu et al.^19^ identified radical **(1)** as the last-step intermediate in the gas-phase abiotic synthesis of uracil from 1*H*-pyrimidin-2-one and hydroxyl radicals. Our experiments indicate that in addition to OH, H atoms can also play an important role in the synthesis of uracil from 1*H*-pyrimidin-2-one. In addition, DHU is a biologically significant pyrimidine derivative found in the loops of transfer RNA (tRNA) and messenger RNA (mRNA).^63^ The reduction of the C5=C6 double bond disrupts aromatic ring character, inducing a puckered conformation that enhances conformational flexibility.^64^ In psychrophilic (cold-adapted) bacteria, this structural modification is particularly beneficial as it prevents excessive RNA rigidity.^65^ While the reported DHU synthesis involves a complex enzyme-mediated process requiring NADPH (nicotinamide adenine dinucleotide phosphate) as a reducing agent and a proton,^66^ our study suggests that abiotic formation via sequential hydrogenation at low temperatures may also occur in the ISM.

Most importantly, our finding that uracil regeneration via hydrogen abstraction of **(1)** and **(2)** is the preferred pathway over further hydrogen addition to form DHU may have profound and highly exciting implications for astrochemistry and the search for life’s origins in space. In the interstellar medium (ISM), particularly in cold molecular clouds and protoplanetary disks, molecules are heavily bombarded by atomic hydrogen, the most abundant radical in the universe. It was generally assumed that once a complex molecule like uracil was formed on a freezing cosmic dust grain, sequential hydrogen addition would rapidly reduce it to 5,6-dihydrouracil and other fully saturated byproducts. If hydrogen abstraction of the radicals to regenerate uracil dominates, atomic hydrogen may consequently act as a recycling agent, resetting the radical to uracil, rather than as a destructive one. This recycling may drastically extend the chemical lifetime of RNA bases in harsh interstellar environments. As uracil has been identified in the carbonaceous chondrites^7–9^ or asteroid,^11^ our discovery may provide an explanation for why these molecules survived the freezing, hydrogen-rich outer solar nebula before being incorporated into parent asteroids.

## 5 Conclusions

In this work, we have successfully recorded and characterized the first infrared spectra of three monohydrogenated uracil radicals: the dominant 5*H*-C_4_H_5_N_2_O_2_ **(1)**, and minor 6*H*-C_4_H_5_N_2_O_2_ **(2)** and, tentatively, 4*OH*-C_4_H_5_N_2_O_2_ **(3)** radicals, generated via hydrogen-atom tunneling reaction with uracil in a *para*-hydrogen matrix at 3.2 K. By pairing matrix-isolation spectroscopy with quantum-chemical vibrational frequency calculations, we achieved excellent agreement between experimental bands and theoretical predictions, resolving a vital gap in the database of transient species associated with prebiotic nucleobases. Under hydrogen-rich conditions, weak features of secondary dihydrogenated product: 5,6*H*-C_4_H_6_N_2_O_2_ (DHU), produced from secondary H addition to **(1)** or **(2)**, were also tentatively identified.

Beyond spectral identification, our mixing ratio estimation revealed that hydrogen addition to the C=C bond prefers the C5 site to form **(1)** rather than the C6 site to form **(2)**, with a branching ratio estimated to be ∼4:1 for radicals **(1)** and **(2)** after IR irradiation. Furthermore, in a hydrogen-rich, dark environment, our real-time temporal profiling revealed a clear anticorrelation between the mixing ratios of uracil and **(1)**, indicating that the transient radicals do not proceed via progressive hydrogenation to saturation. Even though both secondary H-addition (forming DHU) and secondary H-abstraction (regenerating uracil) of the radicals **(1)** and **(2)** are barrierless, the radical-to-parent regeneration pathway is favored over the saturation pathway.

This mechanistic preference may have important implications for chemistry in the interstellar medium (ISM). Rather than rapidly destroying complex pyrimidine rings via full saturation, continuous reactions of uracil with atomic hydrogen may preserve the parent nucleobase structure. This recycling loop may offer a plausible explanation for how uracil can withstand the hostile, hydrogen-rich environments of dark molecular clouds and the outer solar nebula before being exogenously seeded onto early planetary bodies. Further investigations are needed to confirm this possibility.

## Supplementary Material



## Notes

Estimates of mixing ratios (Note 1); comparison of observed and calculated vibrational wavenumbers and IR intensities of uracil; estimates of mixing ratios of uracil and reaction products after each experimental step; comparison of observed and calculated vibrational wavenumbers and IR intensities of 5,6*H*-C_4_H_6_N_2_O_2_ (DHU); vertical excitation wavelengths and oscillator strengths of uracil, 5*H*-C_4_H_5_N_2_O_2_ **(1)**, 6*H*-C_4_H_5_N_2_O_2_ **(2)**, and 4*OH*-C_4_H_4_N_2_O_2_H **(3)**; integrated regions of spectral bands and their IR intensities employed for the estimations of mixing ratios; linear fits of experimental vs calculated vibrational wavenumbers of uracil; comparison of vibrational wavenumbers of uracil in solid *p*-H_2_ with calculated vibrational wavenumbers; geometries of uracil and monohydrogenated, dihydrogenated, H-abstraction products, and transition states in the reaction of H + uracil; IR spectra of uracil/*p*-H_2_ and uracil/Cl_2_/*p*-H_2_ matrix recorded at various stages of experiments; comparison of bands in groups A–D with predicted spectra of possible products of the reactions H + uracil; comparison of bands in group D with DHU in solid Ar, N_2_, and simulated IR stick spectra of DHU; vertical excitation spectra of uracil, 5*H*-C_4_H_5_N_2_O_2_ **(1)**, 6*H*-C_4_H_5_N_2_O_2_ **(2)**, and 4*OH*-C_4_H_5_N_2_O_2_ **(3)**; frontier MO diagrams and transitions of 5*H*-C_4_H_5_N_2_O_2_ **(1)**, 6*H*-C_4_H_5_N_2_O_2_ **(2)**, 4*OH*-C_4_H_5_N_2_O_2_ **(3)**; PES for the formation of DHU and regeneration of uracil (PDF)
