# Supplementary material for: Hydrogenation of Uracil in Solid Para-Hydrogen: Infrared Spectra of Monohydrogenated Radicals (5H‑C4H5N2O2, 6H‑C4H5N2O2, and 4OH-C4H5N2O2) and Their Implications for RNA-Related Chemistry in the Interstellar Medium
Source: J Phys Chem A. 2026 Oct 20;130(43):8597–610. doi: 10.1021/acs.jpca.6c05066 (PMC13726381; doi:10.1021/acs.jpca.6c05066)
Supplement: Supplementary file 1 [file jp6c05066_si_001.pdf]

Supporting Information

Hydrogenation of Uracil in Solid *Para*-Hydrogen: Infrared Spectra of Mono-hydrogenated Radicals (5*H*-$C_4H_5N_2O_2$, 6*H*-$C_4H_5N_2O_2$, and 4*OH*-$C_4H_5N_2O_2$) and Their Implications for RNA-related Chemistry in the Interstellar Medium

Prasad Ramesh Joshi,[a] Manish Kumar Tripathi,[a] and Yuan-Pern Lee*[ab]

[a] Department of Applied Chemistry and Institute of Molecular Science, National Yang Ming Chiao Tung University, Hsinchu 300093, Taiwan

[b] Center for Emergent Functional Matter Science, National Yang Ming Chiao Tung University, Hsinchu 300093, Taiwan

* Corresponding author: yplee@nycu.edu.tw (YPL)

Table of Contents

Note 1. Estimates of mixing ratios S1

Table S1. Comparison of observed and calculated vibrational wavenumbers and IR intensities of uracil S2

Table S2. Estimated mixing ratios of uracil and reaction products after each experimental step S4

Table S3. Comparison of observed and calculated vibrational wavenumbers and IR intensities of 5,6*H*-$C_4H_6N_2O_2$ (DHU) S5

Table S4. Wavelengths and oscillator strengths for vertical excitation of uracil to various excited states predicted with the TD-B3LYP/aug-cc-pVTZ method S6

Table S5. Wavelengths and oscillator strengths for vertical excitation of 5*H*-$C_4H_5N_2O_2$ **(1)** to various excited states predicted with the TD-B3LYP/aug-cc-pVTZ method S7

Table S6. Wavelengths and oscillator strengths for vertical excitation of 6*H*-$C_4H_5N_2O_2$ **(2)** to various excited states predicted with the TD-B3LYP/aug-cc-pVTZ method S8

Table S7. Wavelengths and oscillator strengths for vertical excitation of 4*OH*-$C_4H_5N_2O_2$ **(3)** to various excited states predicted with the TD-B3LYP/aug-cc-pVTZ method S9

Table S8. Integrated regions of spectral bands and their harmonic infrared intensities employed for the estimation of mixing ratios S10

Figure S1. Linear fits of experimental wavenumbers vs. calculated harmonic vibrational wavenumbers of uracil in two spectral ranges S11

Figure S2. Comparison of the experimental infrared spectrum of uracil in solid *p*-H_2 with stick spectra based on scaled harmonic and anharmonic vibrational calculations S12

Figure S3. Geometries of uracil and the three lowest-energy mono-hydrogenated products optimized with the B3LYP/aug-cc-pVTZ method S13

Figure S4. Geometries of other mono-hydrogenated products in the reaction H + uracil optimized with the B3LYP/aug-cc-pVTZ method S14

Figure S5. Geometries of H-abstraction products in the reaction H + uracil optimized with the B3LYP/aug-cc-pVTZ method S16

Figure S6. Geometries of transition states involving hydrogen addition in the reaction H + uracil optimized with the B3LYP/aug-cc-pVTZ method S17

Figure S7. Geometries of transition states involving hydrogen abstraction in the reaction H + uracil optimized with the B3LYP/aug-cc-pVTZ method S18

Figure S8. Geometry of 5,6-dihydrouracil (DHU, 5,6*H*-$C_4H_6N_2O_2$) optimized with the B3LYP/aug-cc-pVTZ method S19

Figure S9. Spectra of a $C_4H_4N_2O_2/Cl_2/p\text{-}H_2$ (1/20/10000) matrix recorded in regions 600−820, 2810−2915, 3400−3495, and 3620−3660 cm^{-1} S20

Figure S10. IR spectra of a $C_4H_4N_2O_2$/*p*-H_2 (1/10000) matrix recorded at various stages of the control experiment S21

Figure S11. Comparison of bands in groups A−C with predicted IR stick spectra of possible products of H + uracil in regions 600−820, 2810−2865, 3400−3495, and 3620−3660 cm^{-1} S22

Figure S12. Comparison of bands in groups A−C with predicted IR stick spectra of other possible H-addition products of the reaction H + uracil S23

Figure S13. Comparison of bands in groups A−C with predicted IR stick spectra of possible H-abstraction products of the reaction H + uracil S24

Figure S14. Comparison of bands in groups A−C with previously reported experimental and calculated spectra of 5,6-dihydrouracil (DHU) S25

Figure S15. Comparison of bands in group D with previously reported experimental and calculated spectra of 5,6-dihydrouracil (DHU) S26

Figure S16. Vertical excitation spectra of uracil and three H-addition radical products S27

Figure S17. The frontier molecular-orbital diagrams and the first two transitions of (a) 5*H*-$C_4H_5N_2O_2$ **(1)** and (b) 6*H*-$C_4H_5N_2O_2$ **(2)**....................S28

Figure S18. The frontier molecular-orbital diagrams and the first two transitions of 4*OH*-$C_4H_5N_2O_2$ **(3)**....................S29

Figure S19. Representative spectra for the identification of bands in group D in a uracil/Cl_2/*p*-H_2 (1/20/10000) matrix S30

Figure S20. Potential-energy scheme predicted for the formation of 5,6-dihydrouracil (DHU) and regeneration of uracil in the reaction H + uracil....................S31

Supporting References S32

Note 1. Estimates of mixing ratios

To estimate the mixing ratios of the species involved in the H + uracil reaction, we employed the methodology introduced by Tam and Fajardo.[1] This approach used two parameters: the absorption pathlength of the matrix and the IR intensities, either experimentally determined or predicted using the B3LYP/aug-cc-pVTZ method. The mixing ratio, denoted by x (in ppm), is calculated using the following equation:

$$x = \frac{2.303 \int \log_{10}(I/I_0)d\nu}{\varepsilon\, l} \times V_m \times 10^6, \qquad \text{(S1)}$$

in which ε is the IR intensity in cm mol^{-1}, $\int \log_{10}(I/I_0)d\nu$ is the experimental integrated absorbance of a specific band, l (in cm) is the IR absorption pathlength through solid p-H_2, and V_m (= 23.16 cm^3 mol^{-1}) is the molar volume of solid p-H_2.[2] Because typically a 2.4 μm filter was placed in the optical path to avoid the reaction of Cl + H_2 during spectral acquisition, the IR absorption pathlength l was determined according to the IR absorption of p-H_2, as described by Fajardo,[3]

$$l = 5.759 \times \int A_{1167}\, d\nu - 0.0075 \qquad \text{(S2)}$$

in which $\int \boldsymbol{A_{1167}\, d\nu}$ represents the integrated absorbance of the band near 1167 cm^{-1}, which corresponds to a pure rotational line of p-H_2.

Due to uncertainties in the calculated IR intensities of each band, the resulting mixing ratios may vary by as much as a factor of 2. To reduce potential errors, we averaged values from multiple spectral bands for each species whenever possible. The integrated regions and associated IR intensities for each species used to estimate mixing ratios are listed in Table S8. The mixing ratio of HCl produced immediately following UV/IR irradiation was employed to estimate the initial mixing ratio of H atoms, assuming that each Cl atom generates one H atom and one HCl molecule via its reaction with H_2, Cl + H_2 (v = 1) → HCl + H. In darkness, the mixing ratio of HCl increases because of the secondary reaction of H atoms with some unreacted Cl atoms.

Table S1. Comparison of Observed and Calculated Vibrational Wavenumbers and IR Intensities of Uracil

mode	sym.	calculations[a]				experiments			
		scaled harmonic[b]		anharmonic		gas	N_2	Ar	*p*-H_2
		ν /cm^{-1}	intensity /km mol^{-1}	ν /cm^{-1}	intensity /km mol^{-1}	ν[c] /cm^{-1}	ν[d] /cm^{-1}	ν[d] /cm^{-1}	ν[e] /cm^{-1}
ν_1	A′	3476	102	3464	86	3484	3468	3485	3479.0
ν_2	A	3435	66	3420	47	3436	3421	3435	3431.3
ν_3	A′	3125	1	3114	1	3124			3132.2
ν_4	A′	3086	2	3068	2				3080.0
$\nu_{21} + \nu_9$				1776	1		1774	1778	1772.5
ν_5	A′	1779	596	1771	515	1756	1761	1764	1764.4
$\nu_{21} + \nu_{10}$				1754	42		1756	1757	1758.3
$\nu_{19} + \nu_{12}$				1741	84		1746	1741	1742.3
$\nu_{21} + \nu_{11}$				1732	120			1733	1732.7
ν_6	A′	1747	778	1730	109			1731	1733.6
$\nu_{17} + \nu_{15}$				1724	75		1735	1728	1729.1
$\nu_{20} + \nu_{12}$				1715	32			1718	1719.0
$\nu_{18} + \nu_{13}$				1705	40	1703	1704	1706	1707.0
$\nu_{17} + \nu_{16}$				1688	179		1707	1698	1699.1
ν_7	A′	1660	68	1638	61	1641	1644	1643	1645.2
$2\nu_{24}$				1521	8	1515	1526	1515	1519.3
ν_8	A′	1484	81	1456	40	1461	1477	1472	1471.7
ν_9	A′	1409	7	1398	14		1405	1399	1406.8
ν_{10}	A′	1393	87	1368	61	1387	1388	1389	1401.3
$\nu_{26} + \nu_{25}$				1382	24			1386	1384.6
ν_{11}	A′	1370	57	1341		1371	1368	1360	1360.9
						1356			
$2\nu_{26}$				1302	32			1313	1323.3
								1304	1313.9
ν_{12}	A′	1215	10	1198	15	1187		1217	1218.0
ν_{13}	A′	1180	105	1158	85	1172	1190	1184	1187.1

$2\nu_{27}$				1113	18			1100	1112.4
ν_{14}	A′	1075	5	1062	2	1082	1069	1073	1074.8
				1064	2			1066	1066.6
ν_{15}	A′	981	7	982	3	990	977	982	984.8
ν_{16}	A′	952	11	942	10	972	962	958	958.6
ν_{17}	A′	757	4	753	3				
ν_{18}	A′	548	5	550	5	545	558	559	
ν_{19}	A′	532	7	535	9		538	536	
ν_{20}	A′	511	22	515	20	512	517	516	
ν_{21}	A′	376	20	386	19	374	393	391	
ν_{22}	A″	974	0	960	0		842	842	
ν_{23}	A″	816	61	810	36	802	811	804	807.1
							809		
ν_{24}	A″	760	35	770	25	757	762	757	759.1
ν_{25}	A″	722	15	720	31	717	724	718	720.5
ν_{26}	A″	677	68	648	77	660	686	662	667.4
							682		
ν_{27}	A″	562	42	558	41		592	551	555.5
ν_{28}	A″	393	31	397	18	395	400		
ν_{29}	A″	159	0	171	0				
ν_{30}	A″	141	2	142	2				

[a]Calculated with the B3LYP/aug-cc-pVTZ method. [b]Harmonic vibrational wavenumber scaled according to $0.9986\,x - 10.8$ for wavenumbers <2500 cm^{-1} and $0.8994\,x + 207.4$ for wavenumbers >2500 cm^{-1}; x is the harmonic vibrational wavenumber. [c]Colarusso, P.; Zhang, K.-Q.; Guo, B.; Bernath, P. F. *Chem. Phys. Lett.* **1997**, 269, 39−48. [d]Barnes, A. J.; Stuckey, M. A.; Le Gall, L. *Spectrochimica Acta. A* **1984**, *40*, 419−431. [e]This work.

Table S2. Estimated Mixing Ratios of Uracil and Reaction Products after Each Experimental Step

species	experimental steps[a]						
	deposition	irrad. 365 nm	irrad. IR	darkness 6 h	darkness (6 + 4) h	irrad. 460 nm	irrad. 399 nm
**(a) H-rich experiment, uracil/Cl_2/*p*-H_2 (1/20/10000)**							
uracil	45.3	44.8	32.6	31.3	31.6	34.4	35.5
		(−1%)[b]	(−27%)	(−4%)	(+1%)	(+9%)	(+3%)
5*H*-$C_4H_5N_2O_2$ **(1)**	0.0	0.3	8.5	9.3	9.0	9.0	2.8
				(+9%)[b]	(−4%)	(~0%)	(−69%)
6*H*-$C_4H_5N_2O_2$ **(2)**	0.0	0.2	3.0	3.0	2.8	2.5	2.4
				(~0%)[b]	(−7%)	(−11%)	(–4%)
4*OH*-$C_4H_5N_2O_2$ **(3)**	0.0	0.07	1.0	1.0	1.0	0.95	0.93
				(~0%)[b]	(~0%)	(−5%)	(−2%)
5,6H-$C_4H_6N_2O_2$	0.0	0.0	0.3	~0.3	~0.5		
(DHU)				(~0%)[b]	(67%)		
**(b) H-deficient experiment, Uracil/Cl_2/*p*-H_2 (1/5/10000)**							
Uracil	47.2	47.0	41.8	41.0	40.7	44.0	44.9
		(~0%)[b]	(−11%)	(−2%)	(−1%)	(+8%)	(+3%)
5*H*-$C_4H_5N_2O_2$ **(1)**	0.0	0.08	4.2	4.6	4.8	1.5	1.5
				(+10%)[b]	(+4%)	(−69%)	(~0%)
6*H*-$C_4H_5N_2O_2$ **(2)**	0.0	0.03	1.1	1.2	1.2	1.1	1.1
				(+9%)[b]	(~0%)	(−8%)	(~0%)

[a]Values are mixing ratios in ppm. [b]The values in parentheses indicate the percentage variations in mixing ratios with respect to that before the experimental step.

Table S3. Comparison of Observed and Calculated Vibrational Wavenumbers and IR Intensities of 5,6*H*-$C_4H_6N_2O_2$ (DHU)

mode	group D, this work			literature	
	p-H_2	B3LYP/aug-cc-pVTZ[a]		solid N_2[b]	solid Ar[b]
	ν /cm^{-1}	ν /cm^{-1}	intensity /km mol^{-1}	ν /cm^{-1}	ν /cm^{-1}
ν_1		3466	49	3467	3479
ν_2		3436	62	3426	3438
ν_3		3012	5		
ν_4		2986	18		
ν_5		2942	8		
ν_6		2898	44		
ν_7	1758.6	1772	390	1758	1753
ν_8		1763	660	1748	1748
ν_9		1512	21	1488	1485
ν_{10}		1463	60	1449	1445
ν_{11}		1447	23	1419	1417
ν_{12}		1410	77	1406	1401
ν_{13}		1402	43	1391	1386
ν_{14}	1362.1	1369	117	1363	1363
ν_{15}		1339	23	1329	1327
ν_{16}		1279	75	1269	1269
ν_{17}		1231	39	1228	1228
ν_{18}	1190.9	1190	102	1190	1190
ν_{19}		1111	57	1114	1115
ν_{20}		1067	1	1065	
ν_{21}		993	3	998	
ν_{22}		940	3	948	946
ν_{23}		926	3	934	
ν_{24}		788	4		793
ν_{25}		757	46	762	758
ν_{26}		706	10	712	707
ν_{27}	670.3	672	78	669	659
ν_{28}		578	11	587	581
ν_{29}		541	37	550	547
ν_{30}		526	54	533	518
ν_{31}		480	3		
ν_{32}		434	19	444	438
ν_{33}		372	21	387	384

[a]Harmonic vibrational wavenumbers scaled according to linear equations $y = 0.9986\,x - 10.8$ and $y = 0.8994\,x + 207.5$ for regions below and above 2500 cm^{-1}, respectively; x is the calculated harmonic vibrational wavenumber. [b]Lapinski, L.; Nowak, M. J.; Bieńko, D. C.; Michalska, D. *Phys. Chem. Chem. Phys.* **2002**, *4*, 1123−1128.

Table S4. Wavelengths and Oscillator Strengths for Vertical Excitation of Uracil to Various Excited States Predicted with the TD-B3LYP/aug-cc-pVTZ Method

$C_4H_4N_2O_2$ (uracil)			
transition number	wavelength /nm	oscillator strength *f*	approximate assignment
1	265.9	0.0000	HOMO−1(α)→LUMO(α)
2	240.9	0.1227	HOMO(α)→LUMO(α)
3	219.4	0.0021	HOMO(α)→LUMO+3(α)
4	215.1	0.0001	HOMO−3(α)→LUMO (α)
5	210.5	0.0321	HOMO−2(α)→LUMO(α)
6	201.3	0.0004	HOMO−1(α)→LUMO+2(α)
7	198.0	0.0253	HOMO−1(α)→LUMO+1(α)
8	192.6	0.1189	HOMO(α)→LUMO+2(α)
9	189.5	0.0040	HOMO(α)→LUMO+3(α)
10	188.7	0.0084	HOMO(α)→LUMO+4(α)
11	185.3	0.0000	HOMO−3(α)→LUMO+2(α)
12	182.1	0.0009	HOMO(α)−2→LUMO+1(α)

Table S5. Wavelength and Oscillator Strength for Vertical Excitation of 5*H*-$C_4H_5N_2O_2$ (1) to Various Excited States Predicted with the TD-B3LYP/aug-cc-pVTZ Method

5*H*-$C_4H_5N_2O_2$ **(1)**			
transition number	wavelength / nm	oscillator strength f	approximate assignment
1	402.0	0.0097	HOMO(α)→LUMO(α)
2	369.2	0.0014	HOMO(α)→LUMO+1(α)
3	299.0	0.0001	HOMO(α)→LUMO+2(α)
4	288.6	0.0002	HOMO(α)→LUMO+3(α)
5	280.3	0.0040	HOMO(α)→LUMO+4(α)
6	274.0	0.0000	HOMO(β)→LUMO(β)
7	256.8	0.0090	HOMO(α)→LUMO+6(α)
8	255.4	0.0000	HOMO−1(α)→LUMO
9	253.6	0.0016	HOMO(α)→LUMO+5(α)
10	242.9	0.0359	HOMO(α)→LUMO+6(α)<br>HOMO−1(β)→LUMO(β)
11	241.4	0.0002	HOMO−2(β)→LUMO(β)
12	237.5	0.0073	HOMO(α)→LUMO+7(α)

Table S6. Wavelength and Oscillator Strength for Vertical Excitation of 6*H*-$C_4H_5N_2O_2$ (2) to Various Excited States Predicted with the TD-B3LYP/aug-cc-pVTZ Method

6*H*-$C_4H_5N_2O_2$ **(2)**			
transition number	wavelength / nm	oscillator strength *f*	approximate assignment
1	552.9	0.0000	HOMO(β)→LUMO(β)
2	420.0	0.0242	HOMO−2(β)→LUMO(β)
3	400.4	0.0029	HOMO−2(β)→LUMO(β)
			HOMO−1(β)→LUMO(β)
4	353.8	0.0001	HOMO−3(β)→LUMO(β)
5	235.5	0.0001	HOMO(α)→LUMO(α)
6	231.2	0.0078	HOMO(α)→LUMO+1(α)
7	221.1	0.0009	HOMO−2(α)→LUMO+1(α)<br>HOMO−1(β)→LUMO+3(β)
8	218.8	0.0000	HOMO−1(α)→LUMO+1(α)
9	208.7	0.0082	HOMO(α)→LUMO+1(α)<br>HOMO−4(β)→LUMO(β)
10	206.7	0.0000	HOMO−1(β)→LUMO+1(β)
11	204.7	0.0002	HOMO−5(β)→LUMO(β)<br>HOMO(β)→LUMO+3(β)
12	204.3	0.0015	HOMO(α)→LUMO+2(α)<br>HOMO−1(β)→LUMO+1(β)

Table S7. Wavelength and Oscillator Strength for Vertical Excitation of 4*OH*-$C_4H_5N_2O_2$ (3) to Various Excited States Predicted with the TD-B3LYP/aug-cc-pVTZ Method

4*OH*-$C_4H_5N_2O_2$ **(3)**			
transition number	wavelength / nm	oscillator strength *f*	approximate assignment
1	558.7	0.0005	HOMO(α)→LUMO(α)
2	514.1	0.0053	HOMO(α)→LUMO+2(α)
3	465.5	0.0017	HOMO−2(β)→LUMO(β)
4	394.8	0.0000	HOMO(α)→LUMO+3(α)
5	360.0	0.0016	HOMO(α)→LUMO+4(α)
6	335.5	0.0011	HOMO(α)→LUMO+5(α)
7	319.6	0.0009	HOMO(α)→LUMO+6(α)
8	314.7	0.0011	HOMO(α)→LUMO+7(α)<br>HOMO(α)→LUMO+6(α)
9	293.3	0.0075	HOMO(β)→LUMO(β)
10	275.8	0.0000	HOMO(α)→LUMO+8(α)
11	272.6	0.0505	HOMO(α)→LUMO+10(α)
12	266.7	0.0000	HOMO(α)→LUMO+9(α)

Table S8. Integrated Regions of Spectral Bands and Their Harmonic Infrared Intensities Employed for the Estimation of Mixing Ratios

species	ν_i	mode description[a]	IR int.[b] / km mol^{-1}	range / cm^{-1}
uracil	ν_{13}	ν_a N1C2N3/ν_a N3C4C5	105	1190.6–1182.9
uracil	ν_{23}	δ C5H (*oop*)/δ C6H (*oop*) (*ip*h)	61	809.0−805.0
uracil	ν_{24}	ring *def* (*oop*)	35	762.3–756.0
5*H*-$C_4H_5N_2O_2$ **(1)**	ν_1	ν N1H	72	3465.4–3459.0
5*H*-$C_4H_5N_2O_2$ **(1)**	ν_{10}	*sci* $C5H_2$/ν_a C5C6N1	39	1433.8–1422.3
5*H*-$C_4H_5N_2O_2$ **(1)**	ν_{21}	ring *def* (*oop*)	57	737.6–733.1
6*H*-$C_4H_5N_2O_2$ **(2)**	ν_2	ν N3H	74	3452.5–3447.8
6*H*-$C_4H_5N_2O_2$ **(2)**	ν_{14}	ν N1C6/ν C2N3 (*ooph*)	110	1143.8–1134.7
6*H*-$C_4H_5N_2O_2$ **(2)**	ν_{24}	ring *def* (*oop*)	32	750.4–746.4
4*OH*-$C_4H_5N_2O_2$ **(3)**	ν_1	ν O4H	89	3652.7–3643.1
4*OH*-$C_4H_5N_2O_2$ **(3)**	ν_8	ν C5=C6/ν N3C4 (*ooph*)	97	1505.5–1497.2
4*OH*-$C_4H_5N_2O_2$ **(3)**	ν_{11}	ν C5=C6/ν C2N3 (*ooph*)	50	1365.8–1355.6
5,6*H*-$C_4H_6N_2O_2$ (DHU)	ν_{18}	ν_a N1C2N3/ν_a N3C4C5	102	1191.9–1190.9
5,6*H*-$C_4H_6N_2O_2$ (DHU)	ν_{27}	δ N3H (*oop*)	78	671.9–669.3

[a]Approximate mode description; *ν*: stretch, δ: bend, *sci*: scissor, *def*: deformation, *opp*: out-of-plane, a: antisymmetric, *ooph*: out-of-phase. [b]Calculated with the B3LYP/aug-cc-pVTZ method.

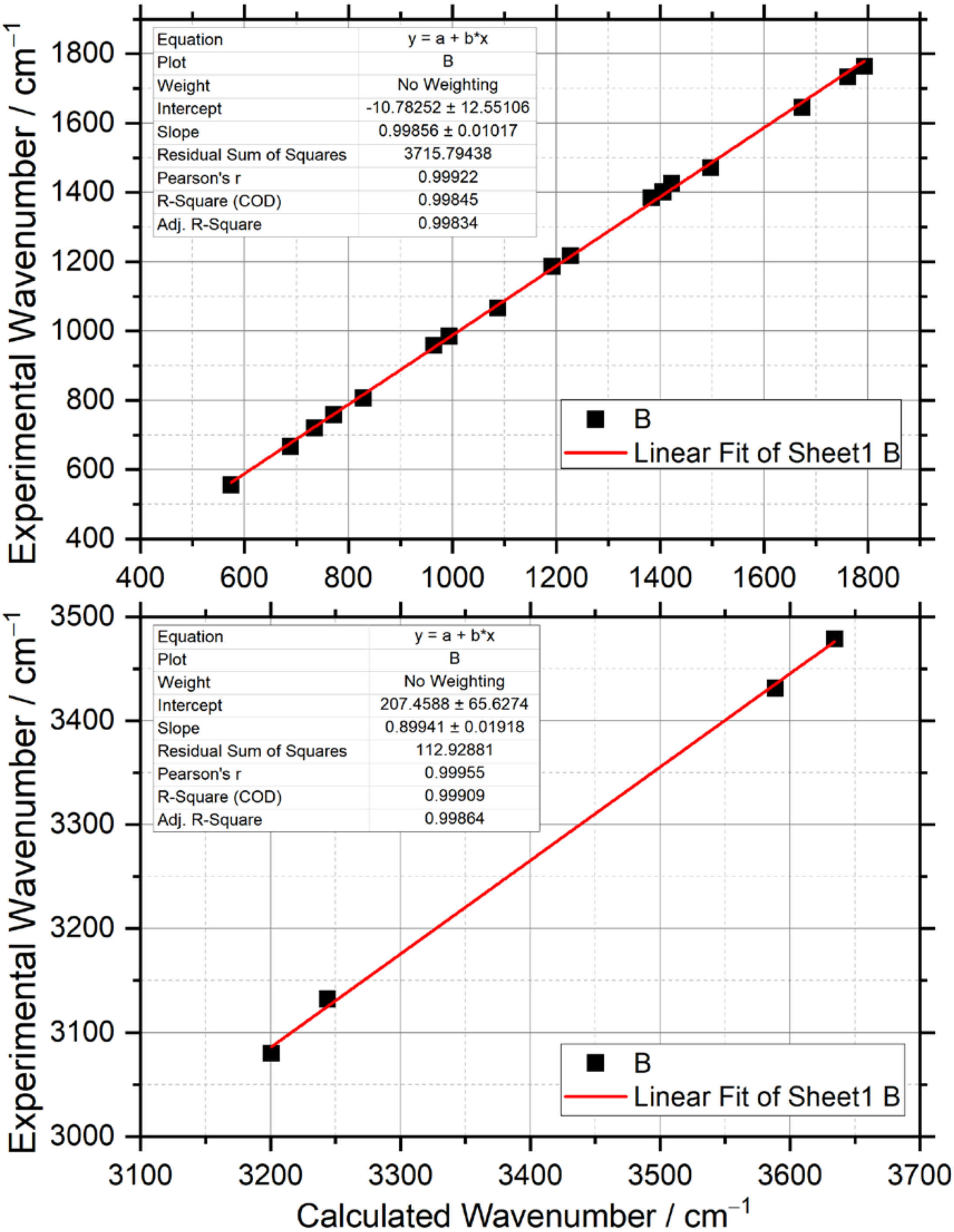

Figure S1. Linear fits of experimental wavenumbers vs. calculated harmonic vibrational wavenumbers of uracil in two spectral ranges. (a) 400−1900 cm^{-1}; (b) 3100−3700 cm^{-1}. Harmonic vibrational wavenumbers were calculated with the B3LYP/aug-cc-pVTZ method. The linear equations obtained on fitting the data for traces (a) and (b) were $y = (0.9986 \pm 0.0102)\ x - (10.8 \pm 12.5)$ and $y = (0.8994 \pm 0.0192)\ x + (207.4 \pm 65.6)$, respectively; y is the observed wavenumber and x is the calculated harmonic vibrational wavenumber.

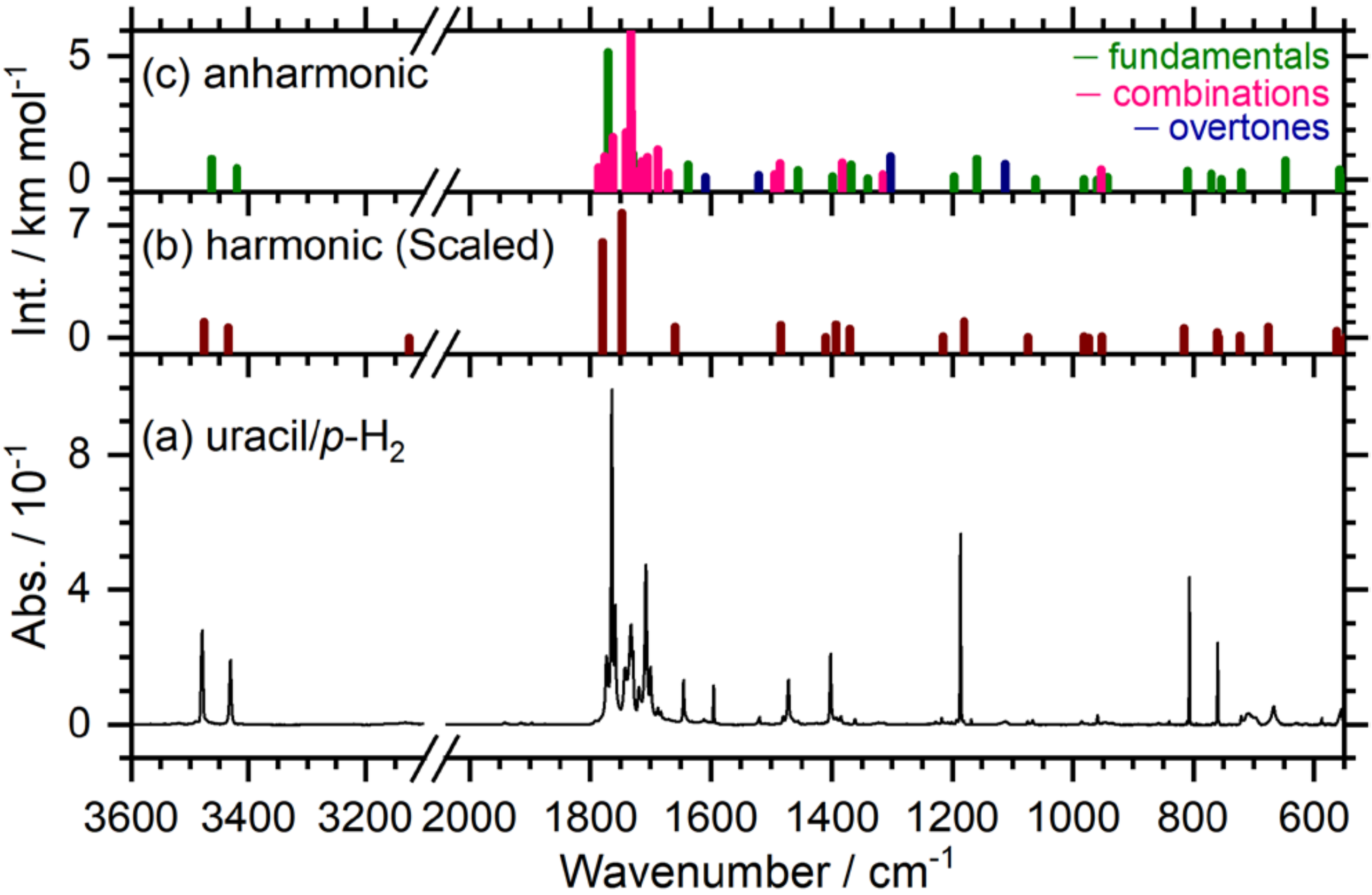

Figure S2. Comparison of the experimental infrared spectrum of uracil in solid *p*-H_2 with stick spectra based on scaled harmonic and anharmonic vibrational calculations. The B3LYP/aug-cc-pVTZ method was employed. Harmonic vibrational wavenumbers were scaled according to $0.9986\,x - 10.8$ for wavenumbers <2500 cm^{-1} and $0.8994\,x + 207.4$ for wavenumbers >2500 cm^{-1}; x is the harmonic vibrational wavenumber. For anharmonic results, combination bands are shown in pink, and overtone bands are shown in blue.

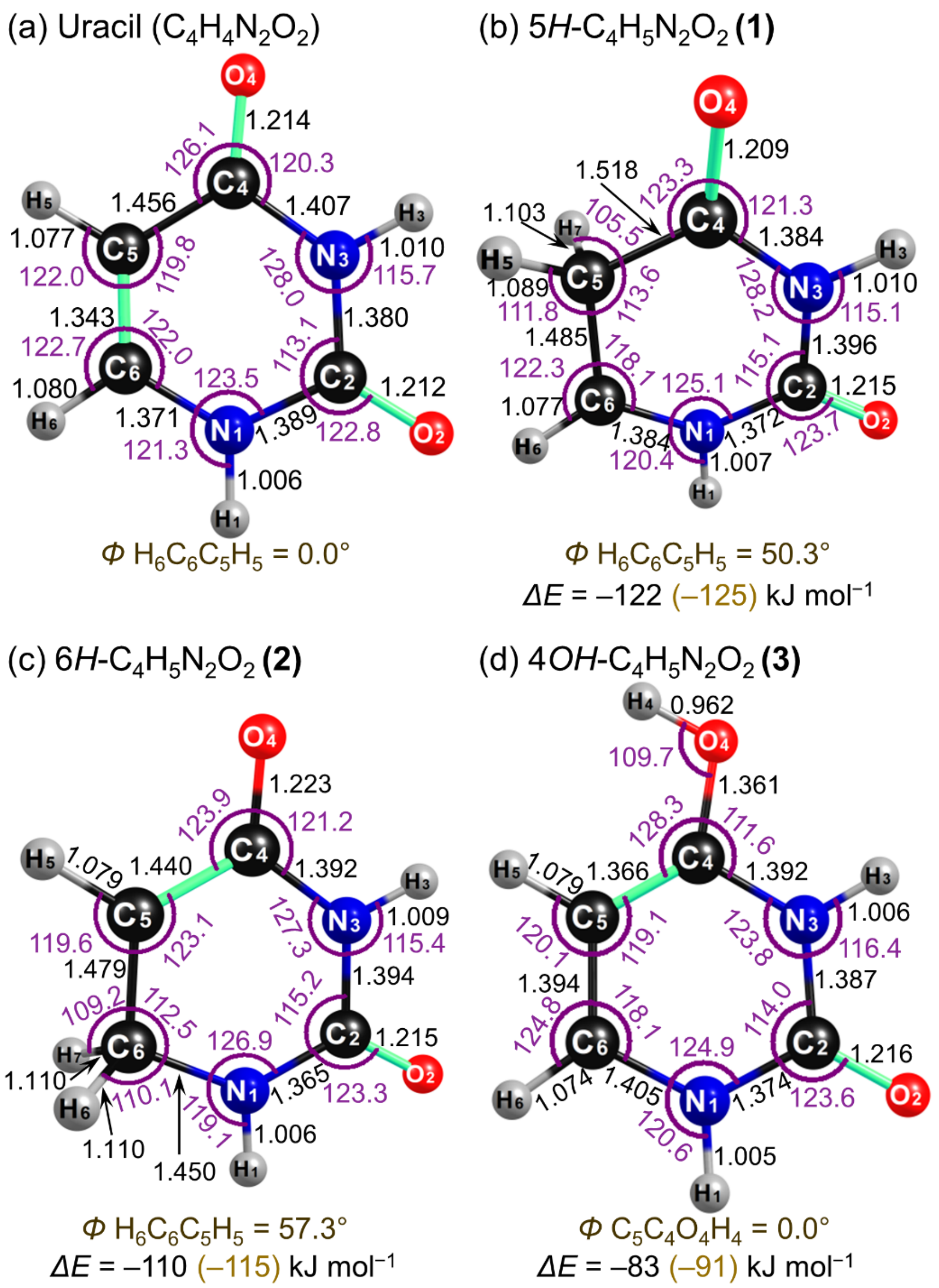

Figure S3. Geometries of uracil and the three lowest-energy mono-hydrogenated products optimized with the B3LYP/aug-cc-pVTZ method. (a) Uracil, (b) 5*H*-$C_4H_5N_2O_2$ **(1)**, (c) 6*H*-$C_4H_5N_2O_2$ **(2)**, and (d) 4*OH*-$C_4H_5N_2O_2$ **(3)**. Bond lengths (black) are in angstrom (Å); bond angles (purple) and dihedral angles Φ are in degrees (°). For (b)−(d), energies (in kJ mol^{-1}) relative to H + uracil, calculated with the CCSD(T)/aug-cc-pVTZ//B3LYP/aug-cc-pVTZ method, are listed in black; those calculated with the B3LYP/aug-cc-pVTZ method are listed in parentheses (olive).

(a) 2*H*-$C_4H_5N_2O_2$

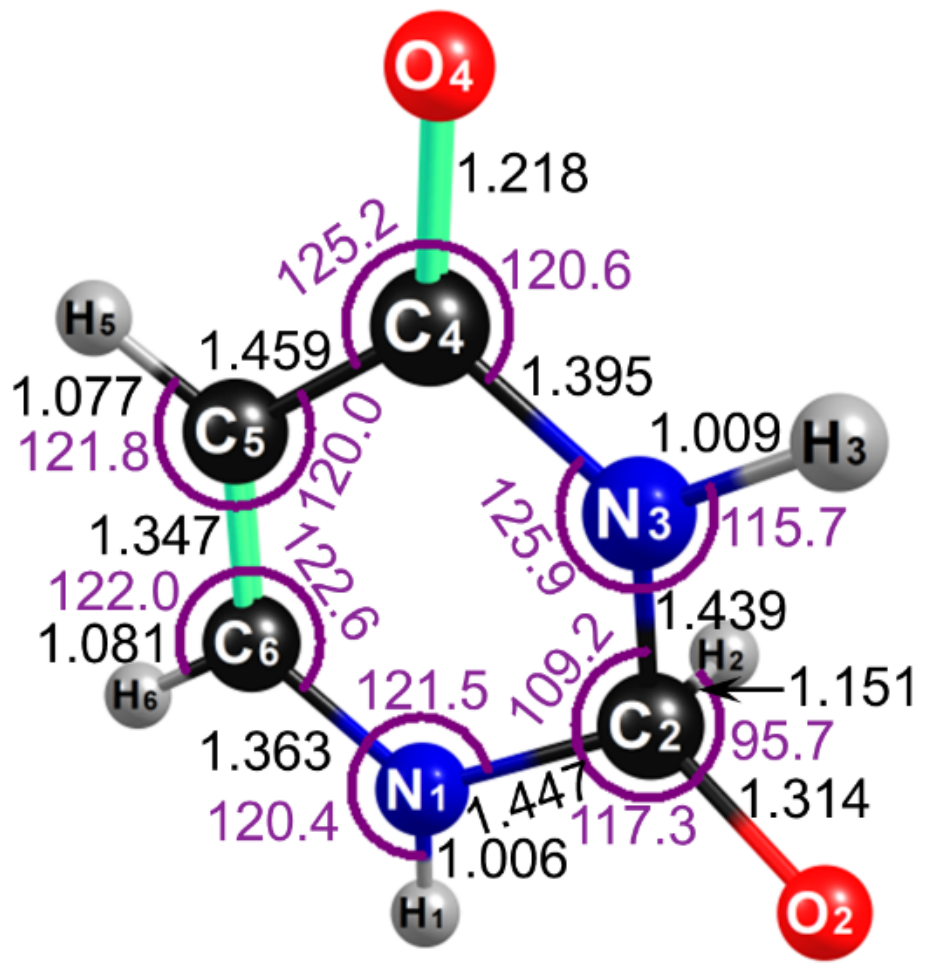

Φ $O_2C_2N_3H_3$ = 32.6°

ΔE = 60 (44) kJ mol^{-1}

(b) 4*H*-$C_4H_5N_2O_2$

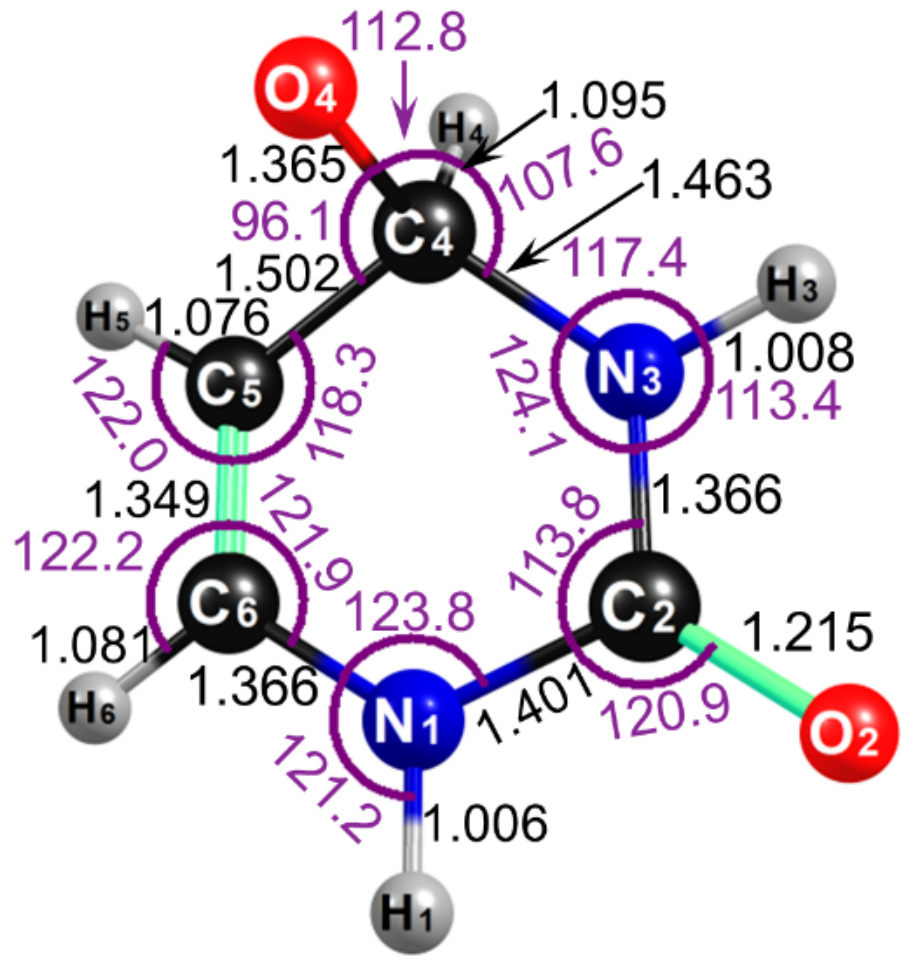

Φ $O_4C_4C_5H_5$ = 77.2°

ΔE = 19 (10) kJ mol^{-1}

(c) 1*H*-$C_4H_5N_2O_2$

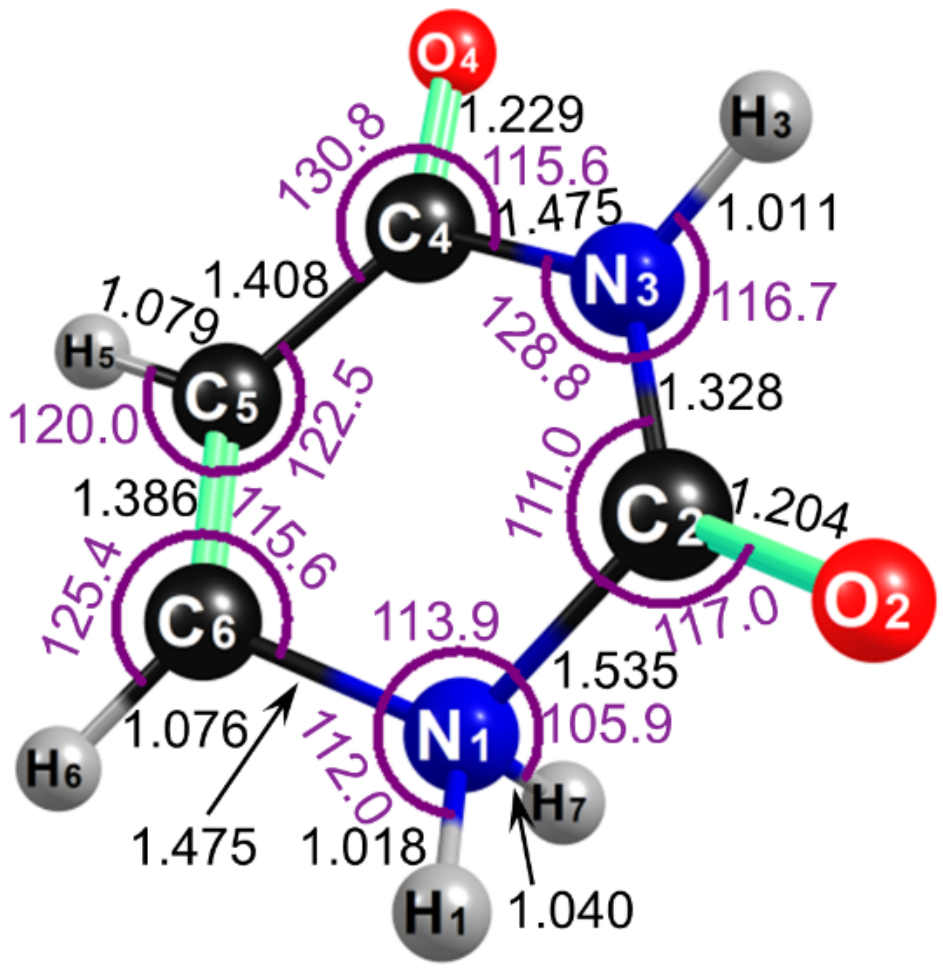

Φ $H_1N_1C_2O_2$ = 22.9°

ΔE = 66 (46) kJ mol^{-1}

(d) 3*H*-$C_4H_5N_2O_2$

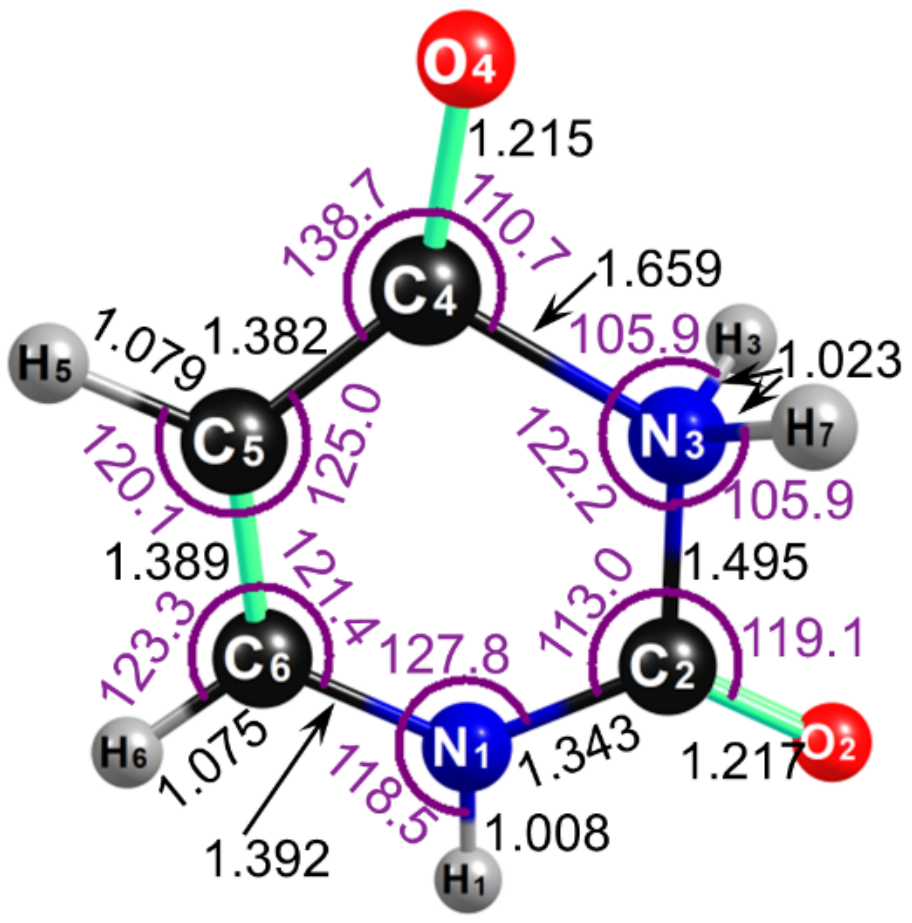

Φ $O_2C_2N_3H_3$ = 56.6°

ΔE = 28 (9) kJ mol^{-1}

(e) 2*OH*-$C_4H_5N_2O_2$

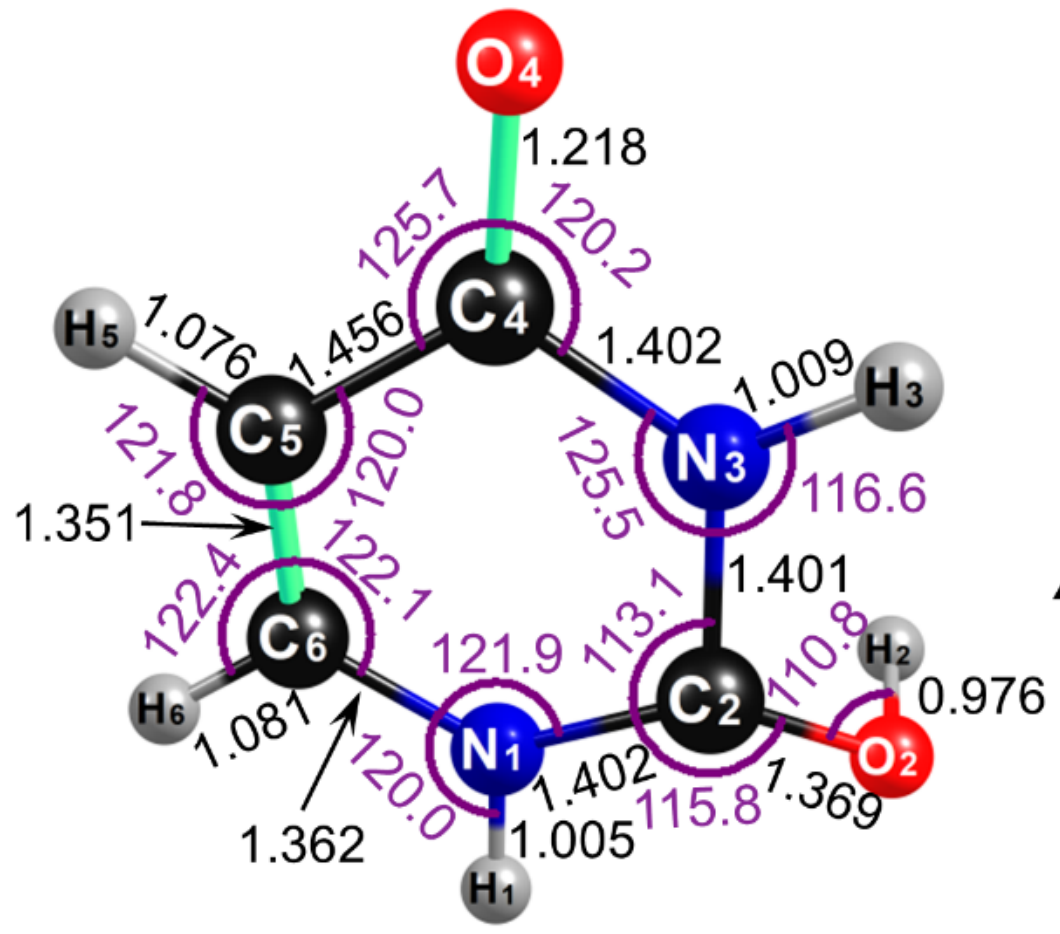

Φ $N_1C_2O_2H_2$ = 77.9°

ΔE = –18 (–22) kJ mol^{-1}

Figure S4. Geometries of other mono-hydrogenated products in the reaction H + uracil optimized with the B3LYP/aug-cc-pVTZ method. (a) 2*H*-$C_4H_5N_2O_2$, (b) 4*H*-$C_4H_5N_2O_2$, (c) 1*H*-$C_4H_5N_2O_2$, (d) 3*H*-$C_4H_5N_2O_2$, and (e) 2*OH*-$C_4H_5N_2O_2$. Bond lengths (black) are in angstrom (Å); bond angles (purple) and dihedral angles Φ are in degrees (°). Energies (in kJ mol^{-1}) relative to H + uracil, calculated with the CCSD(T)/aug-cc-pVTZ//B3LYP/aug-cc-pVTZ method, are listed in black; those calculated with the B3LYP/aug-cc-pVTZ method are listed in parentheses (olive).

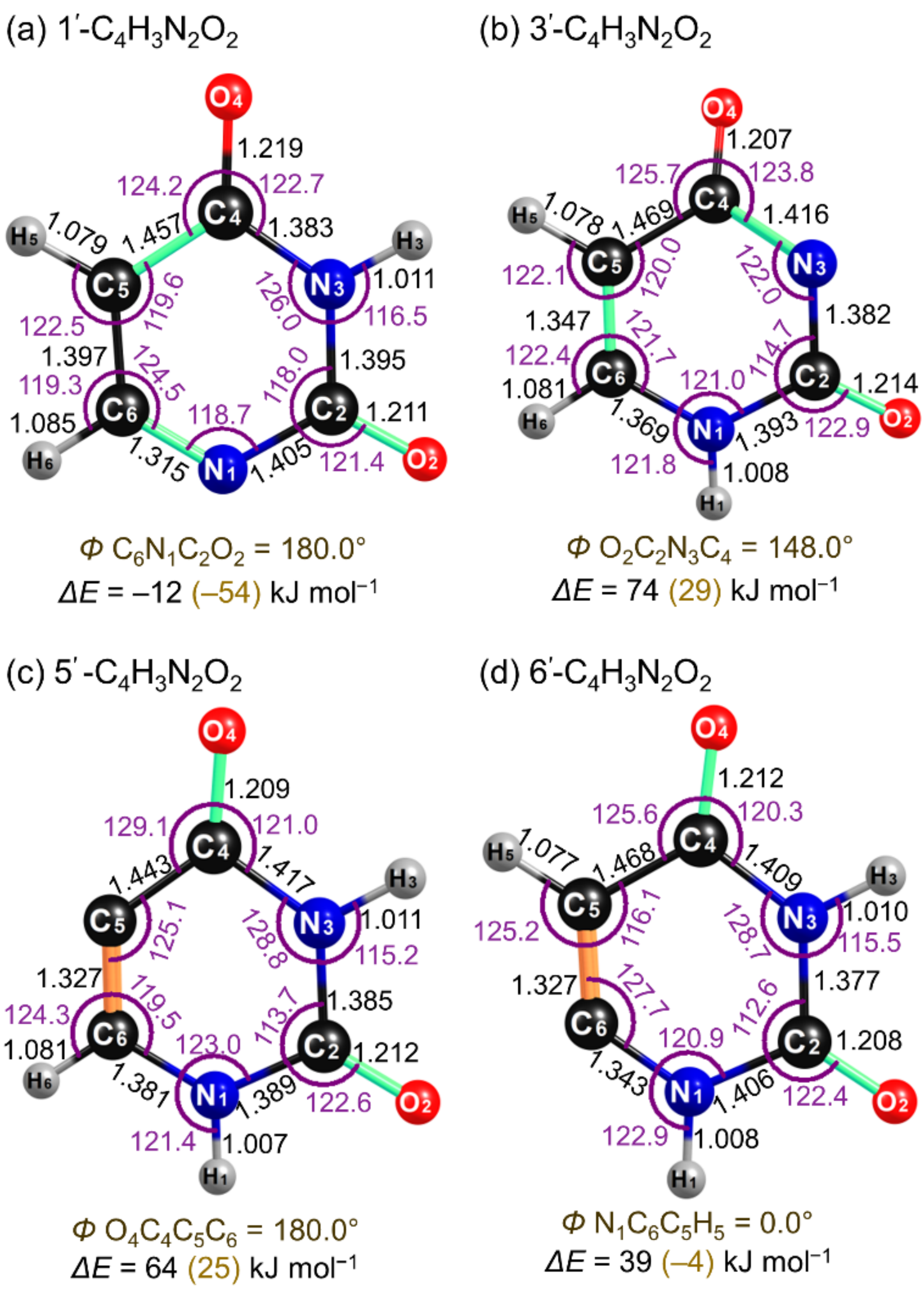

Figure S5. Geometries of H-abstraction products in the reaction H + uracil optimized with the B3LYP/aug-cc-pVTZ method. (a) 1′-$C_4H_3N_2O_2$, (b) 3′-$C_4H_3N_2O_2$, (c) 5′-$C_4H_3N_2O_2$, and (d) 6′-$C_4H_3N_2O_2$. The prime indicates the site of H-abstraction. Bond lengths (black) are in angstrom (Å); bond angles (purple) and dihedral angles Φ are in degrees (°). Energies (in kJ mol^{-1}) relative to H + uracil − H_2 calculated with the CCSD(T)/aug-cc-pVTZ//B3LYP/aug-cc-pVTZ method are listed in black; those with the B3LYP/aug-cc-pVTZ method are listed in parentheses (olive).

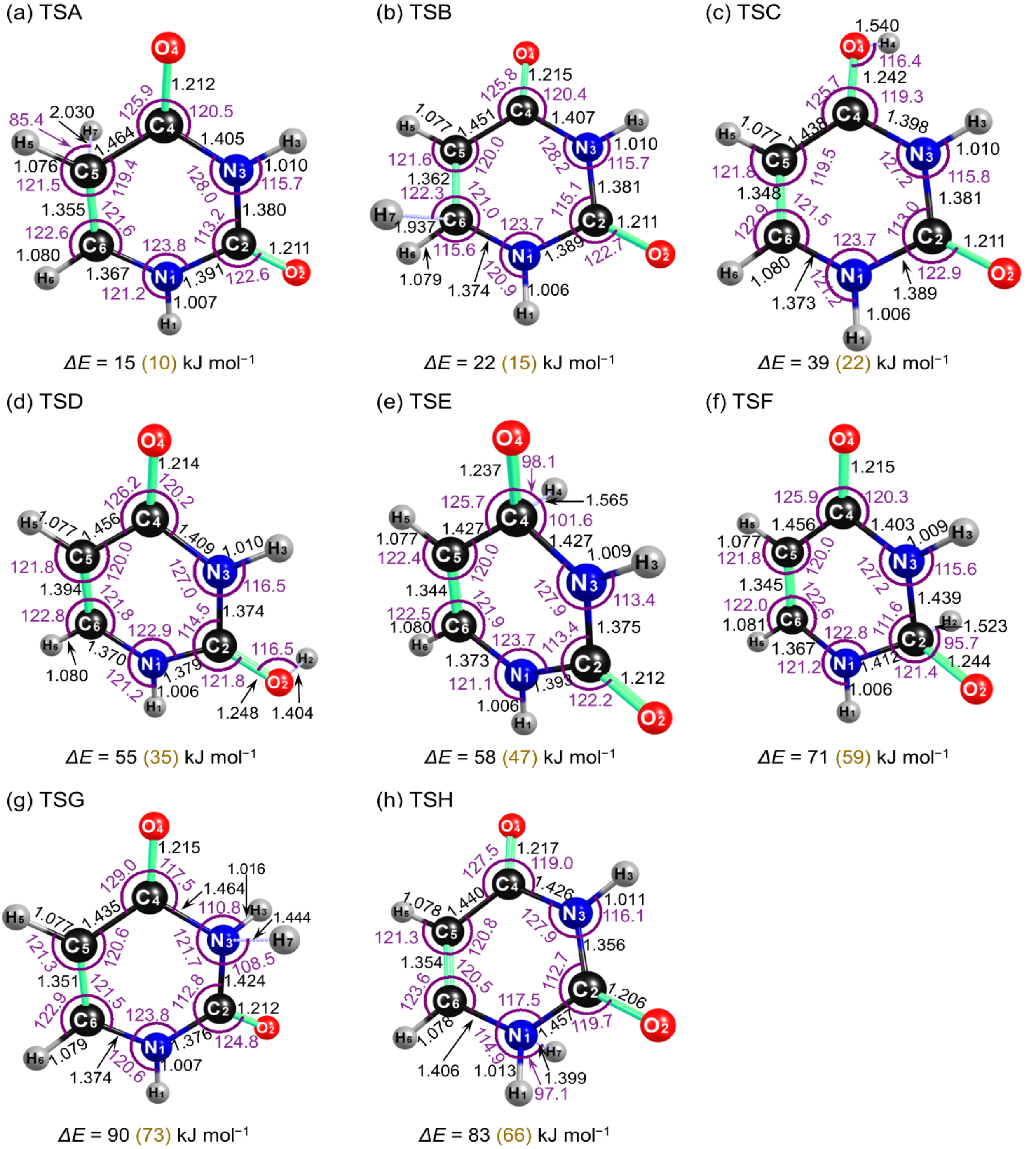

Figure S6. Geometries of transition states involving hydrogen addition in the reaction H + uracil optimized with the B3LYP/aug-cc-pVTZ method. (a) TSA, (b) TSB, (c) TSC, (d) TSD, (e) TSE, (f) TSF, (g) TSG, and (h) TSH. Bond lengths (black) are in angstrom (Å); bond angles (purple) are in degrees (°). Energies (in kJ mol^{-1}) relative to H + uracil calculated with the CCSD(T)/aug-cc-pVTZ//B3LYP/aug-cc-pVTZ method are listed in black; those with the B3LYP/aug-cc-pVTZ method are listed in parentheses (olive).

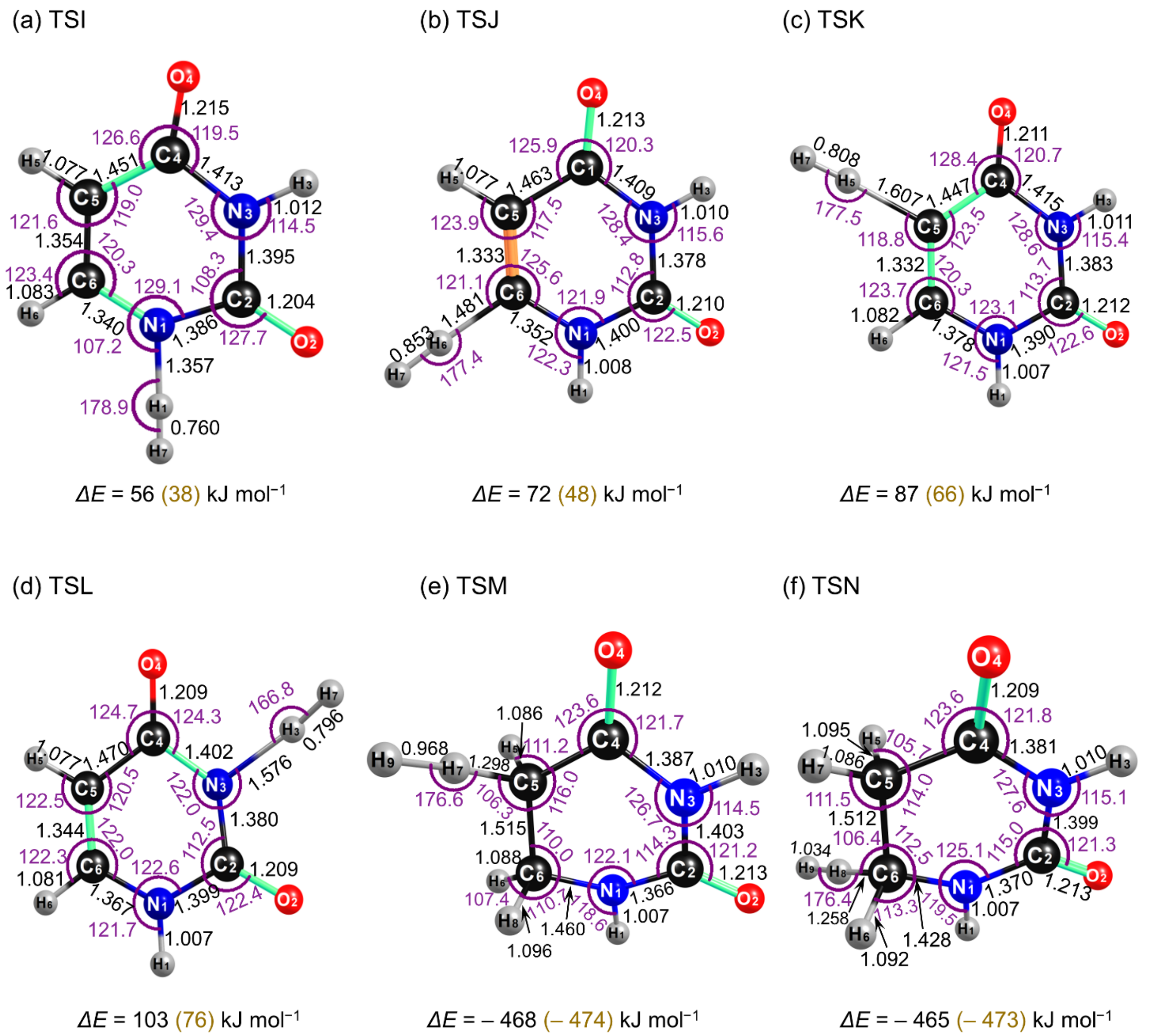

Figure S7. Geometries of transition states involving hydrogen abstraction in the reaction H + uracil optimized with the B3LYP/aug-cc-pVTZ method. (a) TSI, (b) TSJ, (c) TSK, (d) TSL, (e) TSM, and (f) TSN. Bond lengths (black) are in angstrom (Å), and bond angles (purple) are in degrees (°). Energies (in kJ mol^{-1}) relative to H + uracil calculated with the CCSD(T)/aug-cc-pVTZ//B3LYP/aug-cc-pVTZ method are listed in black; those with the B3LYP/aug-cc-pVTZ method are listed in parentheses (olive).

5,6H-$C_4H_6N_2O_2$ (DHU)

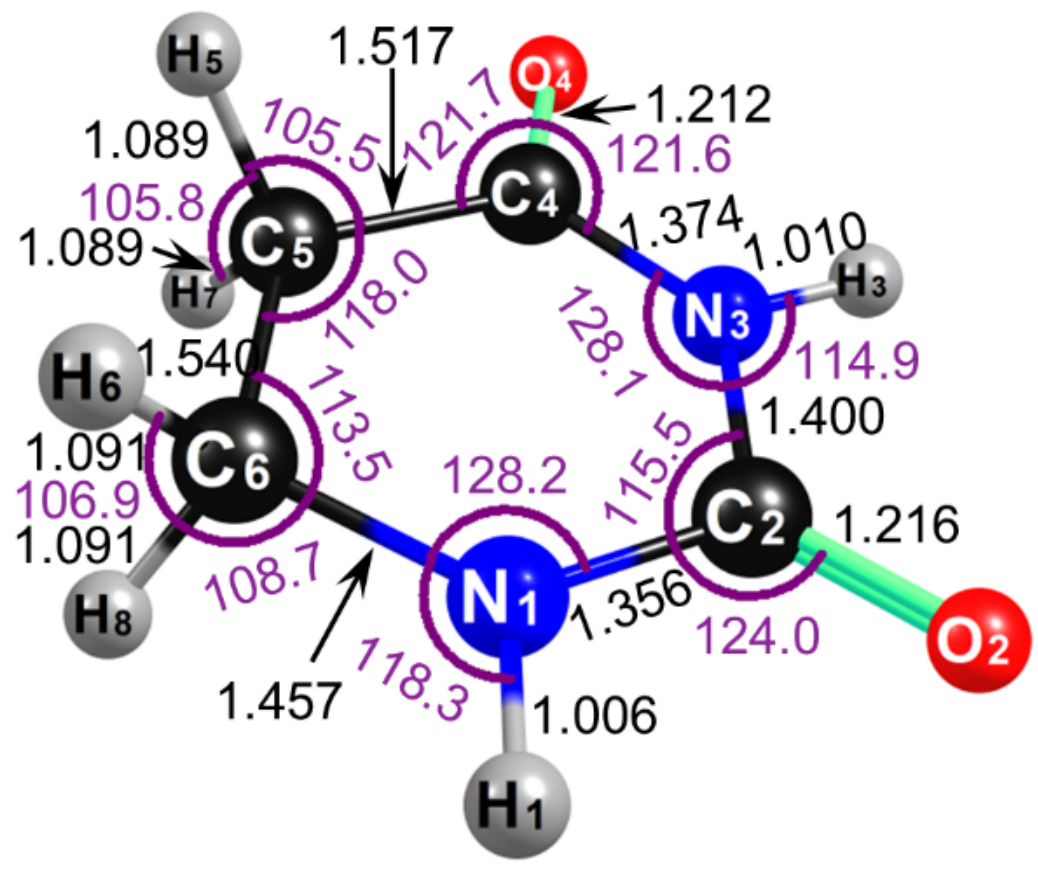

Φ $H_5C_5C_6H_8$ = 116.7°
Φ $H_6C_6N_1H_1$ = 58.1°
Φ $O_4C_4C_5H_7$ = 55.9°

Figure S8. Geometry of 5,6-dihydrouracil (DHU, 5,6H-$C_4H_6N_2O_2$) optimized with the B3LYP/aug-cc-pVTZ method. Bond lengths (black) are in angstrom (Å); bond angles (purple) are in degrees (°).

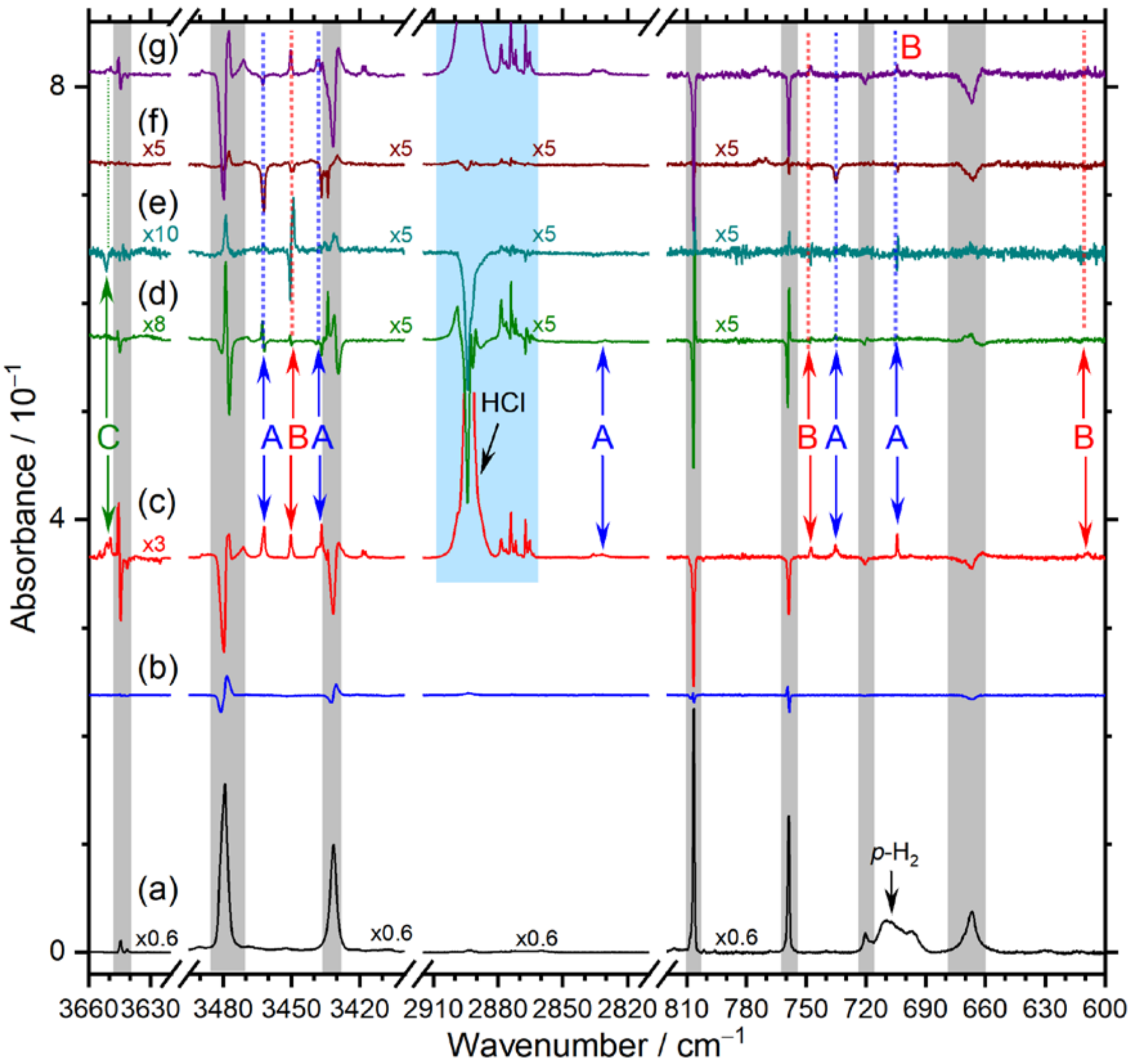

Figure S9. Spectra of a $C_4H_4N_2O_2/Cl_2/p\text{-}H_2$ (1/20/10000) matrix recorded in regions 600−820, 2810−2915, 3400−3495, and 3620−3660 cm^{-1} at various stages of the experiment. (a) Spectrum recorded after deposition at 3.2 K for 6 h. (b) Difference spectrum after photolysis at 365 nm for 30 min. (c) Difference spectrum after additional full IR irradiation for 1 h. (d) Difference spectrum after maintaining the matrix in darkness for 10 h. (e) Difference spectrum after secondary irradiation at 460 nm for 20 min. (f) Difference spectrum after further secondary irradiation at 399 nm for 20 min. (g) Processed spectrum of (c) + 0.57×(f); the scaling factor was determined by diminishing the feature near 1157.2 cm^{-1} in (c). Bands in groups A−C are indicated with blue, red, and green arrows and labels, respectively. Areas shaded in gray indicate spectral regions that are interfered with by uracil absorption.

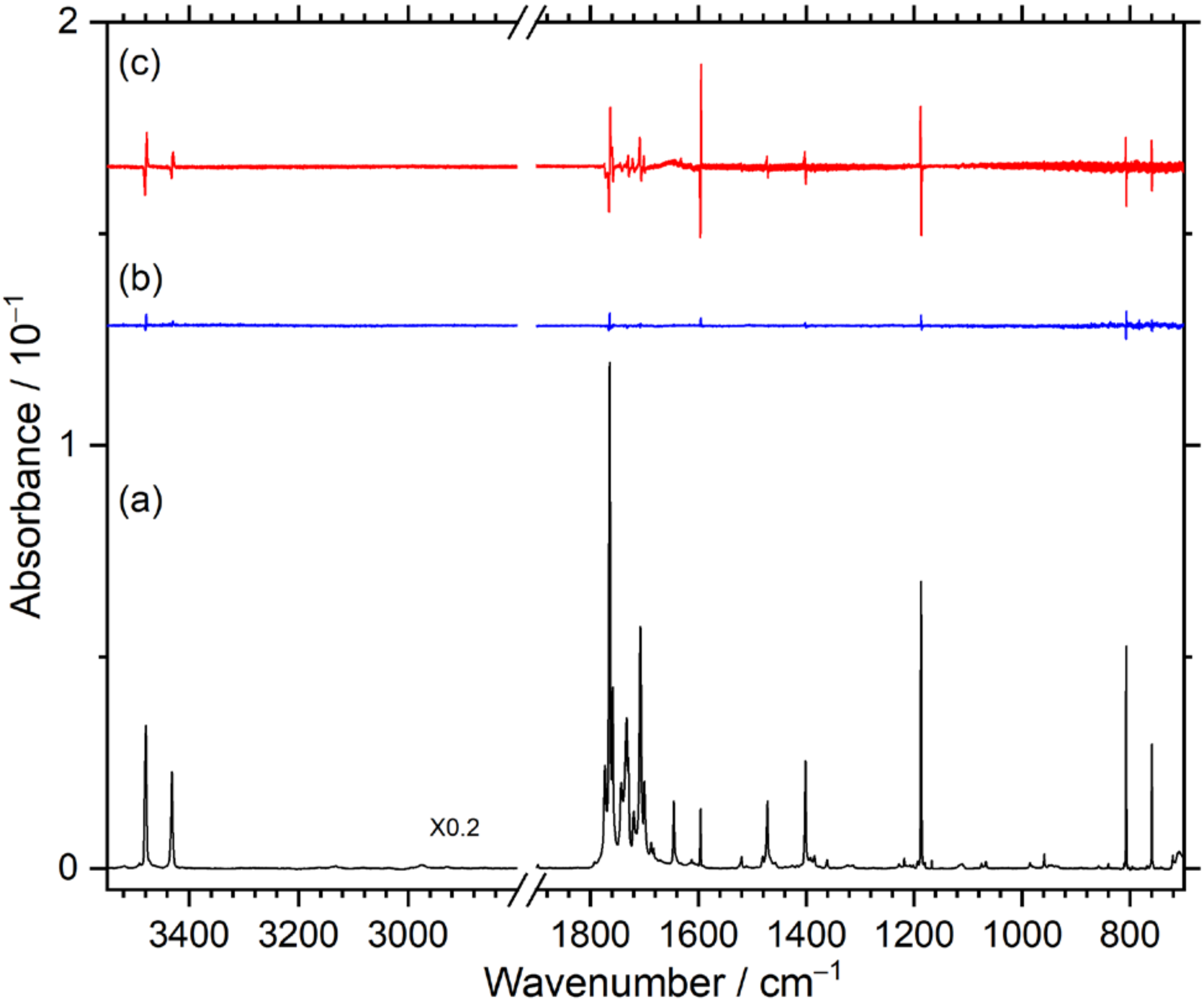

Figure S10. IR spectra of a $C_4H_4N_2O_2$/*p*-H_2 (1/10000) matrix recorded at various stages of the control experiment. (a) Spectrum recorded after deposition at 3.2 K for 6 h. (b) Difference spectrum after photolysis at 365 nm for 30 min. (c) Difference spectrum after further full-IR irradiation for 1 h.

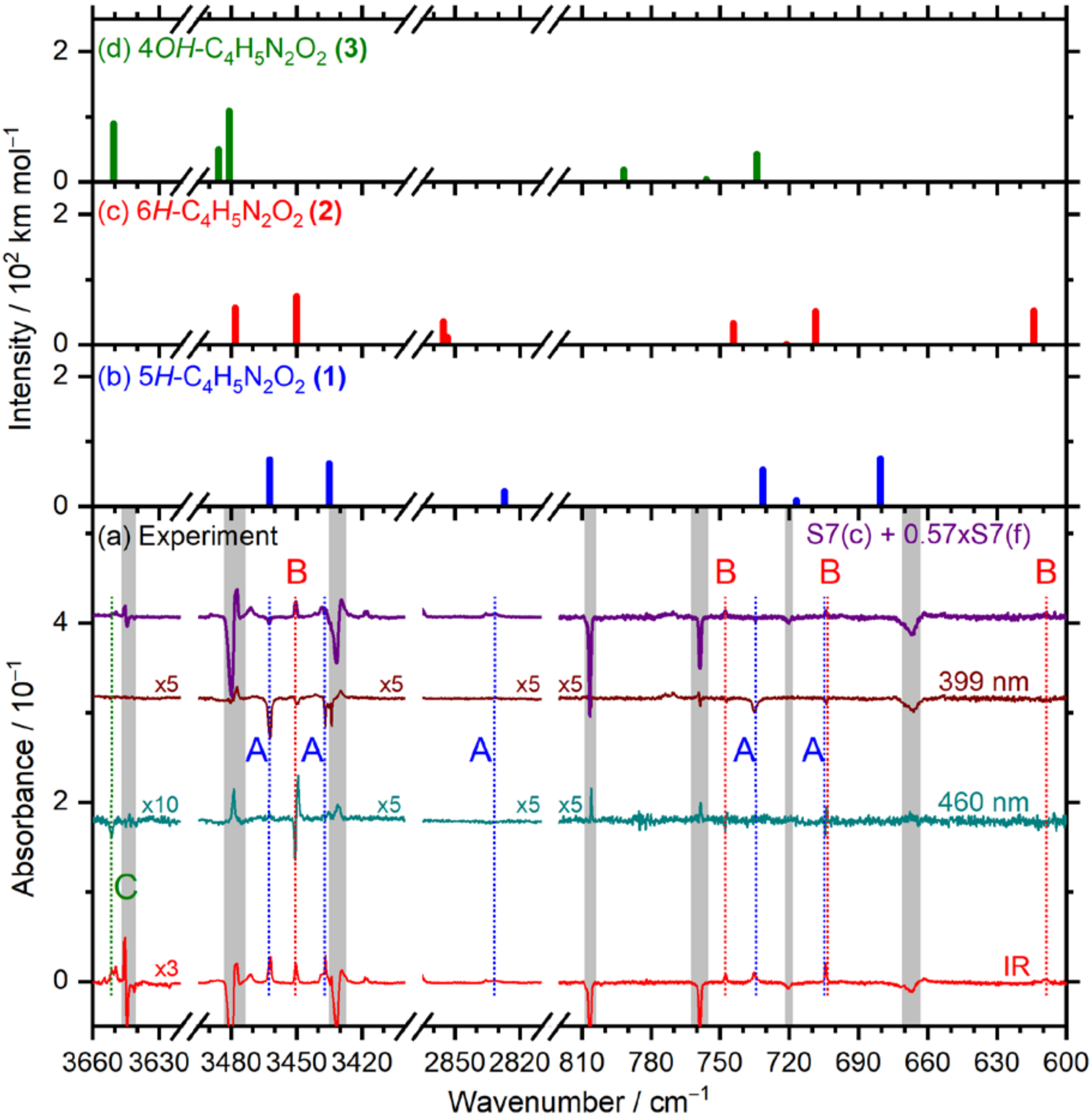

Figure S11. Comparison of bands in groups A−C with predicted IR stick spectra of possible products of H + uracil in regions 600−820, 2810−2865, 3400−3495, and 3620−3660 cm^{-1}. (a) Experimental spectra: red, cyan, brown, and purple traces are taken from Figures S9c, S9e, S9f, and S9g, respectively. Bands in groups A−C are indicated with blue, red, and green arrows and labels, respectively. The simulated IR stick spectra are (b) 5*H*-$C_4H_5N_2O_2$ **(1)**, (c) 6*H*-$C_4H_5N_2O_2$ **(2)**, and (d) 4*OH*-$C_4H_5N_2O_2$ **(3)**, according to scaled harmonic vibrational wavenumbers and IR intensities calculated with the B3LYP/aug-cc-pVTZ method. The areas shaded in gray indicate spectral regions that are interfered with by uracil absorption.

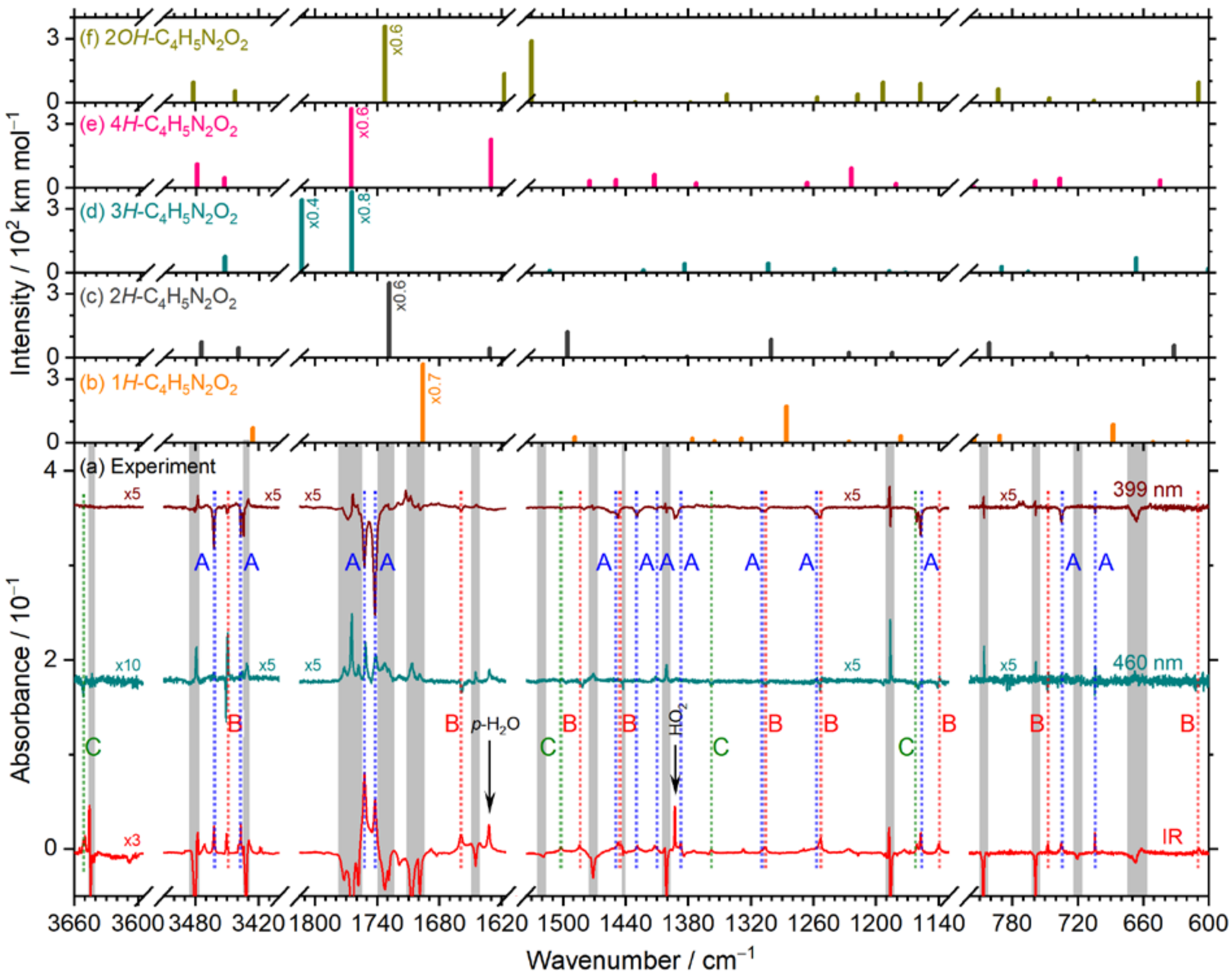

Figure S12. Comparison of bands in groups A–C with predicted IR stick spectra of other possible H-addition products of the reaction H + uracil. (a) Experimental spectra: red, cyan, and brown traces are taken from Figures 3c and S9c, Figures 3e and S9e, and Figures 3f and S9f, respectively. Bands in groups A, B, and C are indicated with blue, red, and green arrows and labels. The simulated IR stick spectra are (b) 1*H*-$C_4H_5N_2O_2$, (c) 2*H*-$C_4H_5N_2O_2$, (d) 3*H*-$C_4H_5N_2O_2$, (e) 4*H*-$C_4H_5N_2O_2$, and (f) 2*OH*-$C_4H_5N_2O_2$, according to scaled harmonic vibrational wavenumbers and IR intensities calculated with the B3LYP/aug-cc-pVTZ method. The areas shaded in gray indicate spectral regions that are interfered with by uracil absorption.

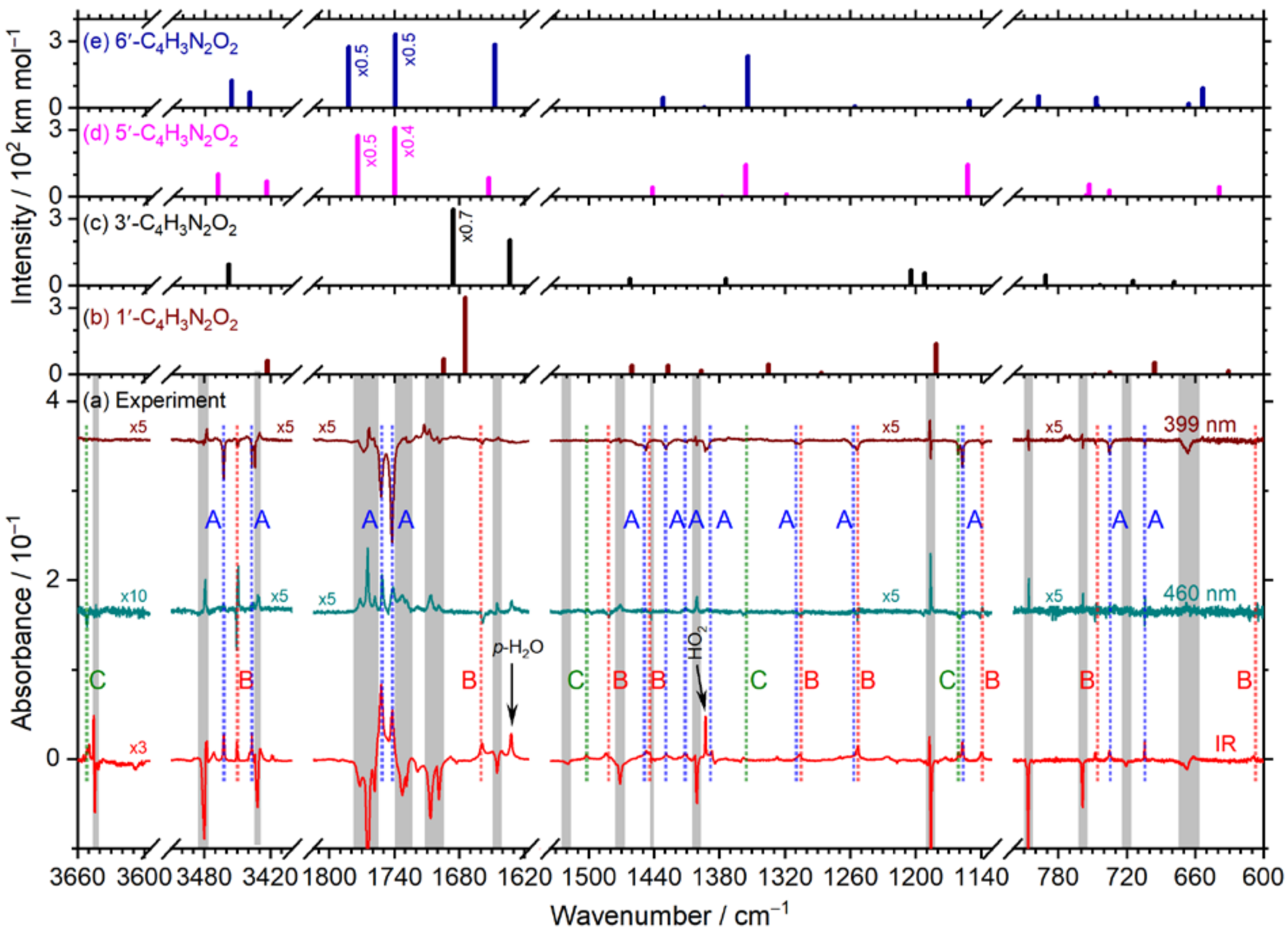

Figure S13. Comparison of bands in groups A–C with predicted IR stick spectra of possible H-abstraction products of the reaction H + uracil. (a) Experimental spectra: red, cyan, and brown traces are taken from Figures 3c and S9c, Figures 3e and S9e, and Figures 3f and S9f, respectively. Bands in groups A, B, and C are marked with blue, red, and green arrows and labels. The simulated IR stick spectra are (b) 1'-$C_4H_3N_2O_2$, (c) 3'-$C_4H_3N_2O_2$, (d) 5'-$C_4H_3N_2O_2$, and (e) 6'-$C_4H_3N_2O_2$ according to scaled harmonic vibrational wavenumbers and IR intensities calculated with the B3LYP/aug-cc-pVTZ method. The areas shaded in gray indicate spectral regions that are interfered with by uracil absorption.

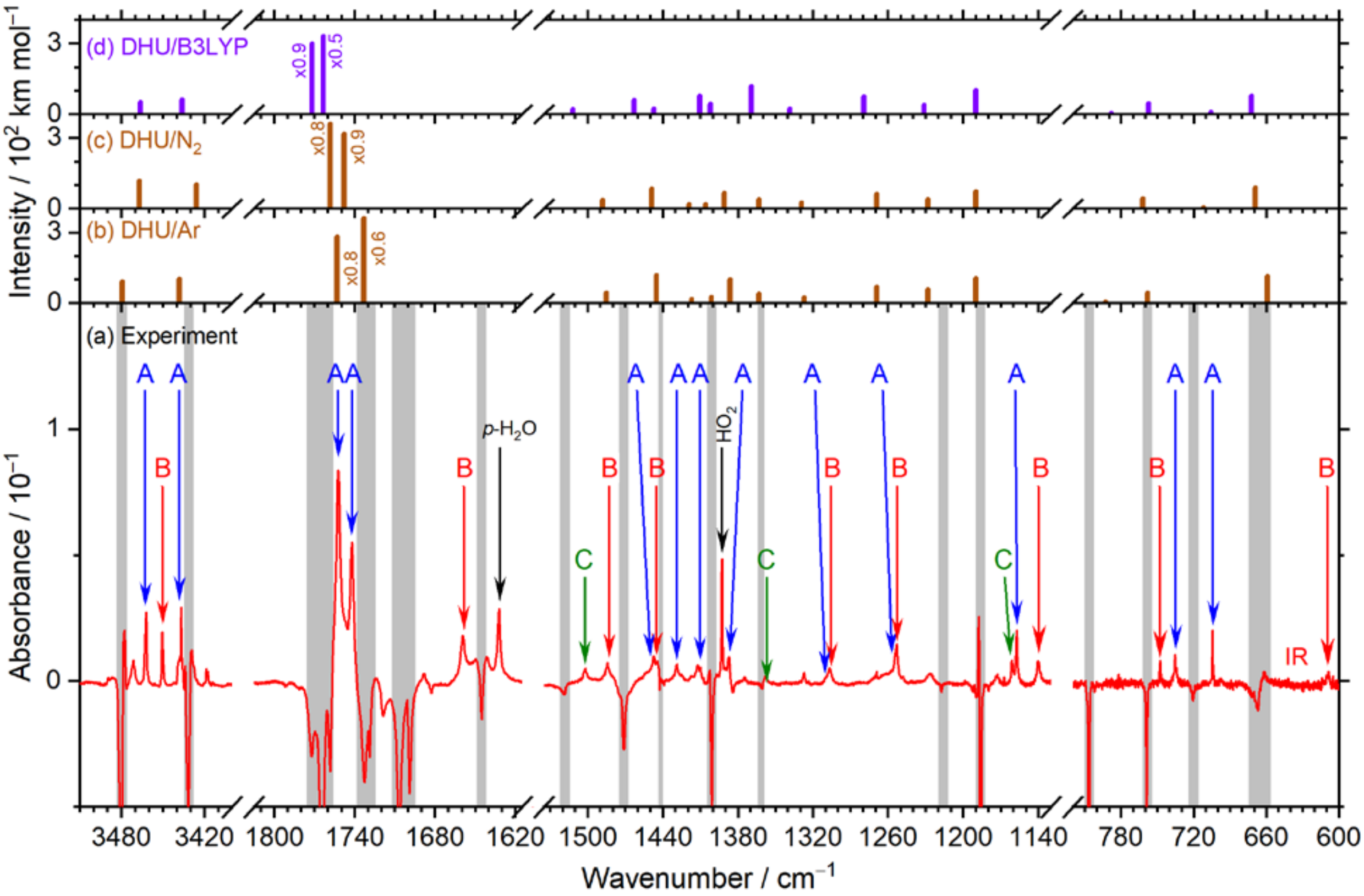

Figure S14. Comparison of bands in groups A−C with previously reported experimental and calculated spectra of 5,6-dihydrouracil (DHU). (a) The difference spectrum after additional full IR irradiation for 1 h is reproduced from Figures 3c and S9c of the H-rich experiment with uracil/Cl_2/*p*-H_2 (1/20/10000). Bands in groups A−C are indicated with blue, red, and green arrows and labels, respectively. The previously reported IR stick spectra of DHU in solid Ar (b) and solid N_2 (c) are from [b]Lapinski, L.; Nowak, M. J.; Bieńko, D. C.; Michalska, D. *Phys. Chem. Chem. Phys.* **2002**, *4*, 1123−1128. (d) The scaled harmonic vibrational stick spectrum of DHU, calculated with the B3LYP/aug-cc-pVTZ method. The areas shaded in gray indicate spectral regions that are interfered with by uracil absorption.

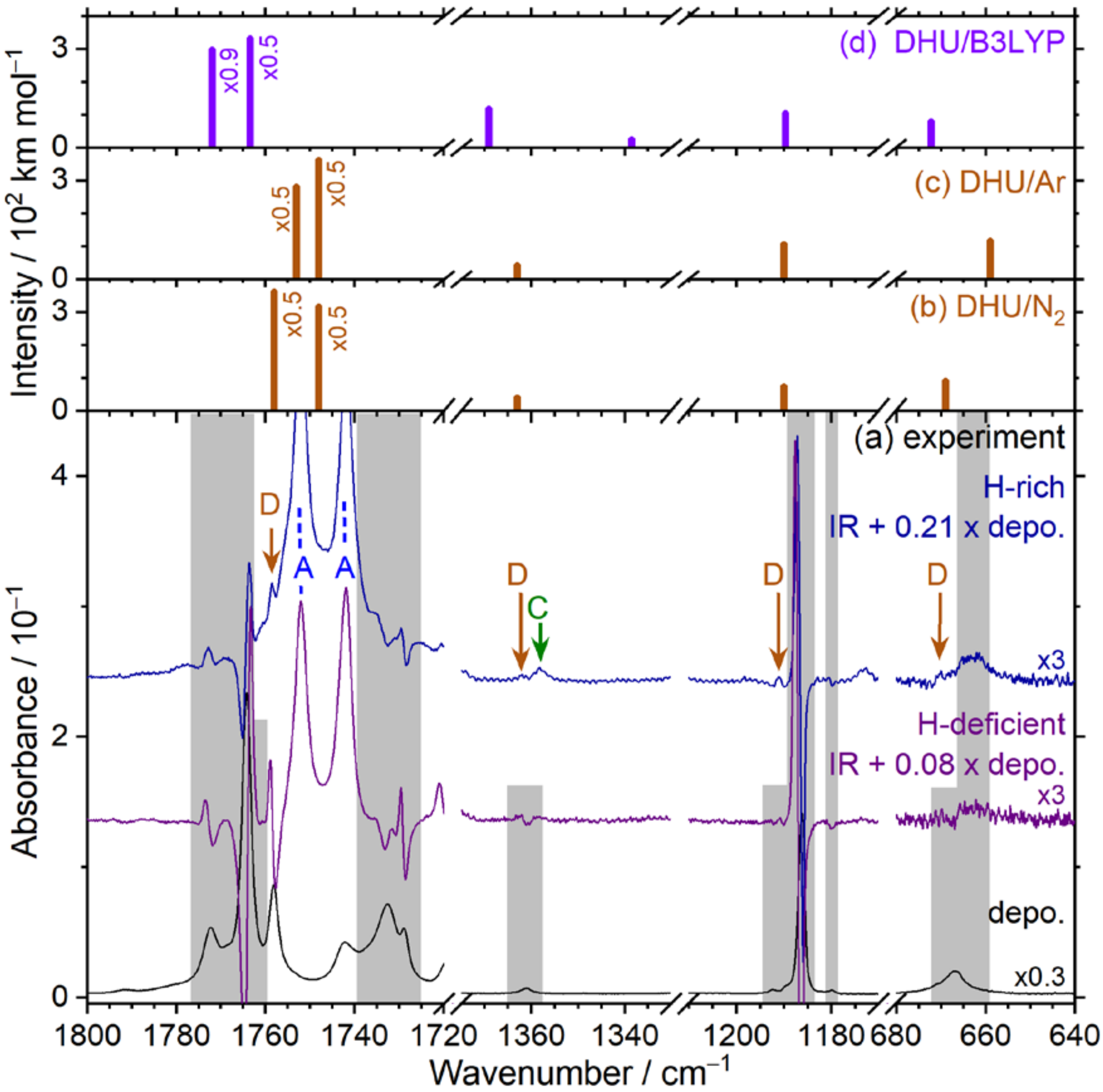

Figure S15. Comparison of bands in group D with previously reported experimental and calculated spectra of 5,6-dihydrouracil (DHU). (a) The lower trace is from Figures 3a and S9a, the middle trace is the processed spectrum Figure 3c + 0.08 × Figure 3a and Figure S9c + 0.08 × Figure S9a obtained from the H-deficient experiment with uracil/Cl_2/*p*-H_2 (1/6/10000), and the upper trace is the processed spectrum Figure 3c + 0.21 × Figure 3a and Figure S9c + 0.21 × Figure S9a obtained from the H-rich experiment with uracil/Cl_2/*p*-H_2 (1/20/10000). Bands in groups A, C, and D are marked with blue, green, and brown arrows and labels, respectively. The previously reported IR stick spectra of DHU in solid Ar (b) and solid N_2 (c) are from Lapinski, L.; Nowak, M. J.; Bieńko, D. C.; Michalska, D. *Phys. Chem. Chem. Phys.* **2002**, *4*, 1123–1128. (d) The scaled harmonic vibrational stick spectrum of DHU, calculated with the B3LYP/aug-cc-pVTZ method. The areas shaded in gray indicate spectral regions that are interfered with by uracil absorption.

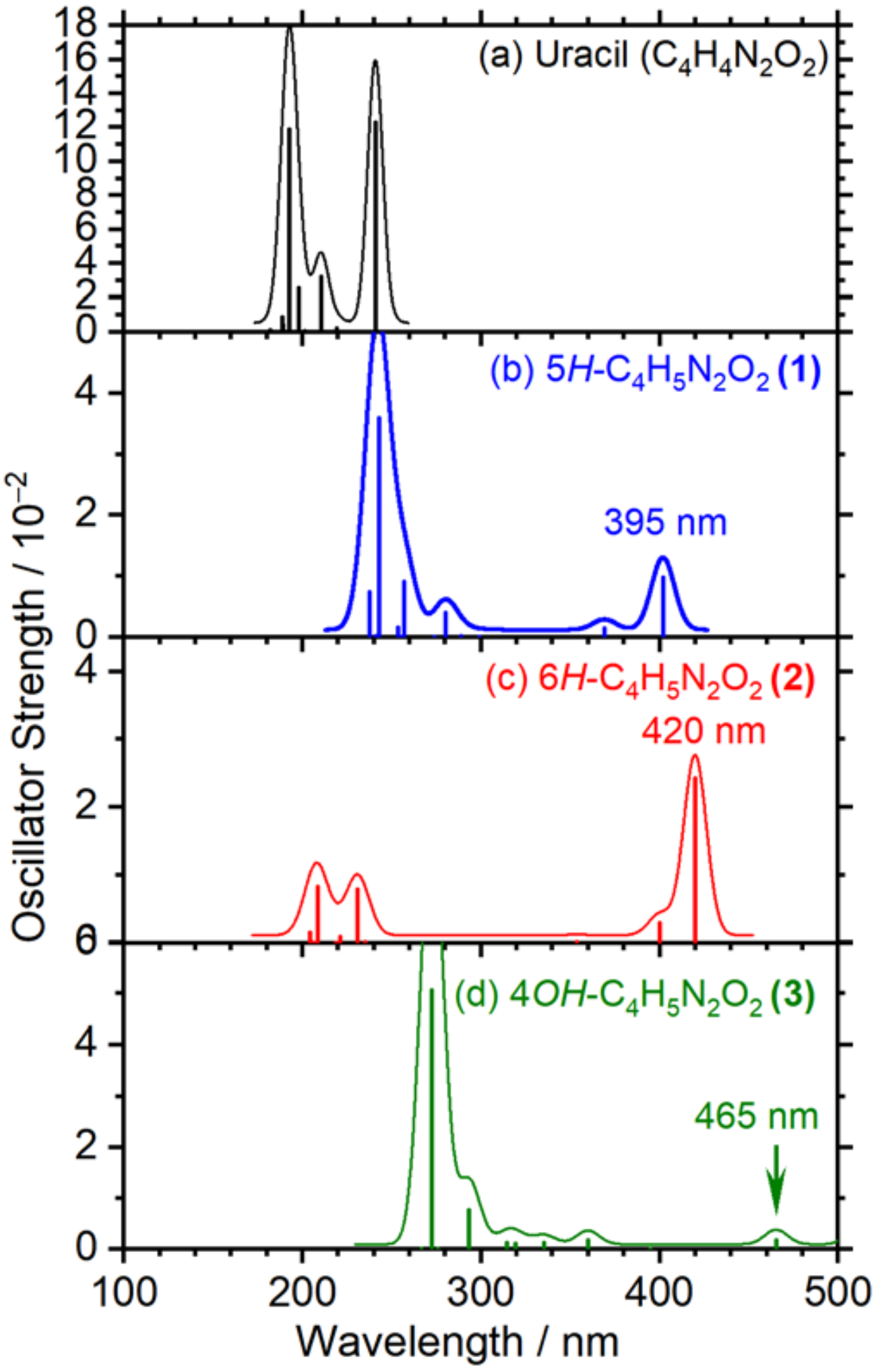

Figure S16. Vertical excitation spectra of uracil and three H-addition radical products. (a) uracil, (b) 5*H*-$C_4H_5N_2O_2$ **(1)**, (c) 6*H*-$C_4H_5N_2O_2$ **(2)**, and (d) 4*OH*-$C_4H_5N_2O_2$ **(3)**. The stick spectra were calculated using the TD-B3LYP/aug-cc-pVTZ method; the UV spectra were convoluted with a full width at half maximum (FWHM) of 20 nm.

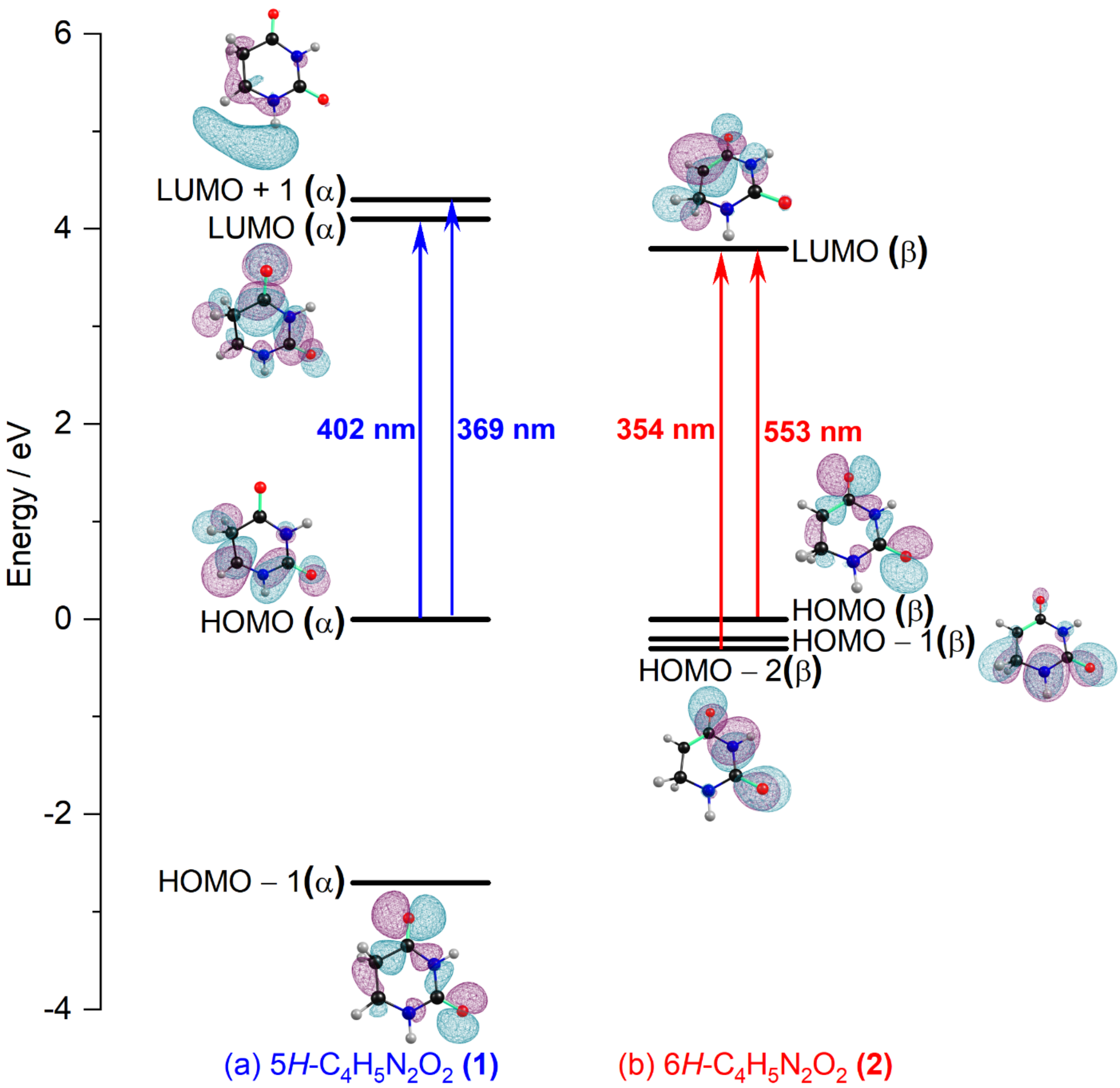

Figure S17. The frontier molecular-orbital diagrams and the first two transitions of (a) 5*H*-$C_4H_5N_2O_2$ **(1)** and (b) 6*H*-$C_4H_5N_2O_2$ **(2)**. The isovalue contours (0.03 a.u.) were calculated with the TD-B3LYP/aug-cc-pVTZ method.

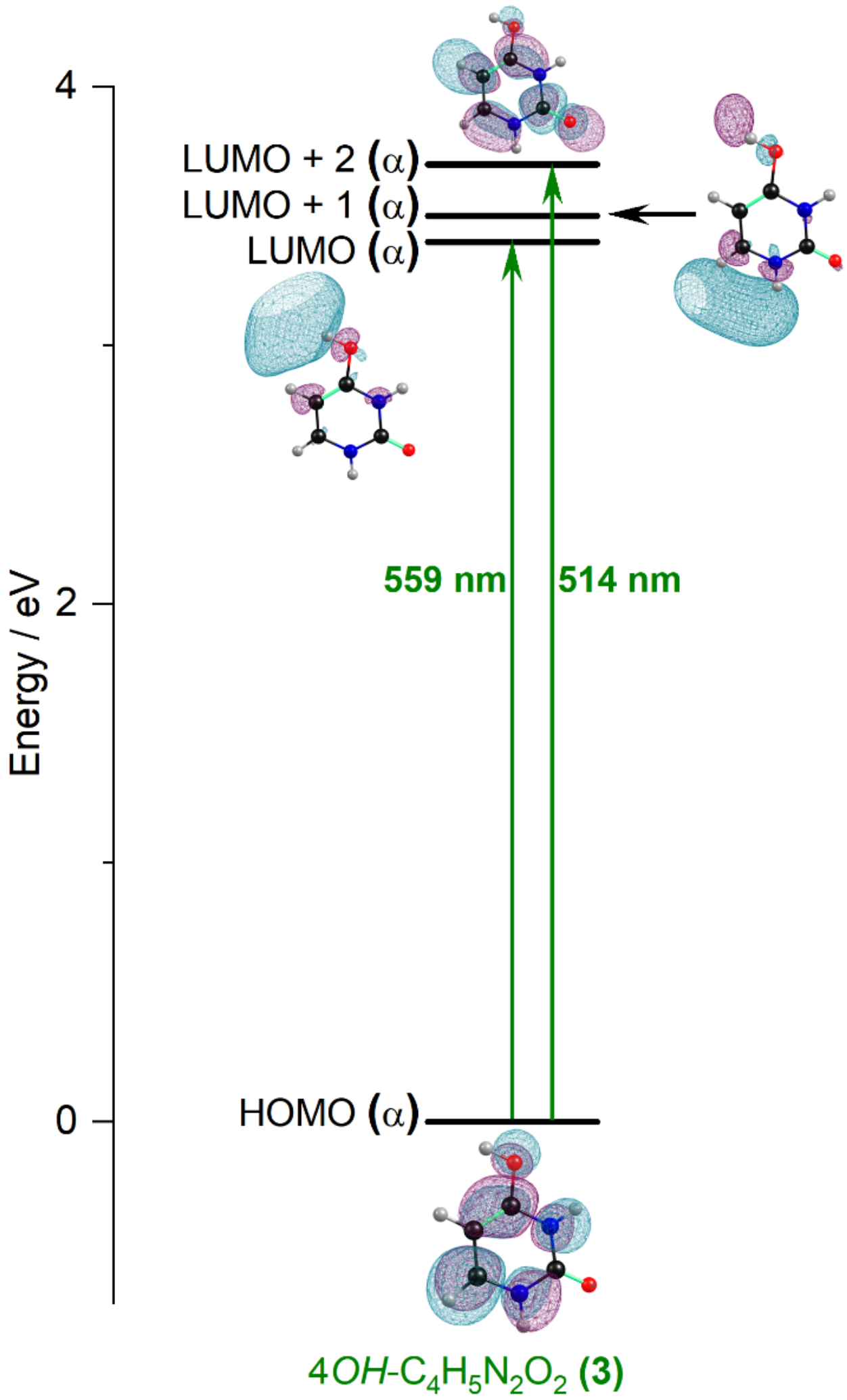

Figure S18. The frontier molecular-orbital diagrams and the first two transitions of 4*OH*-$C_4H_5N_2O_2$ **(3)**. The isovalue contours (0.03 a.u.) were calculated with the TD-B3LYP/aug-cc-pVTZ method.

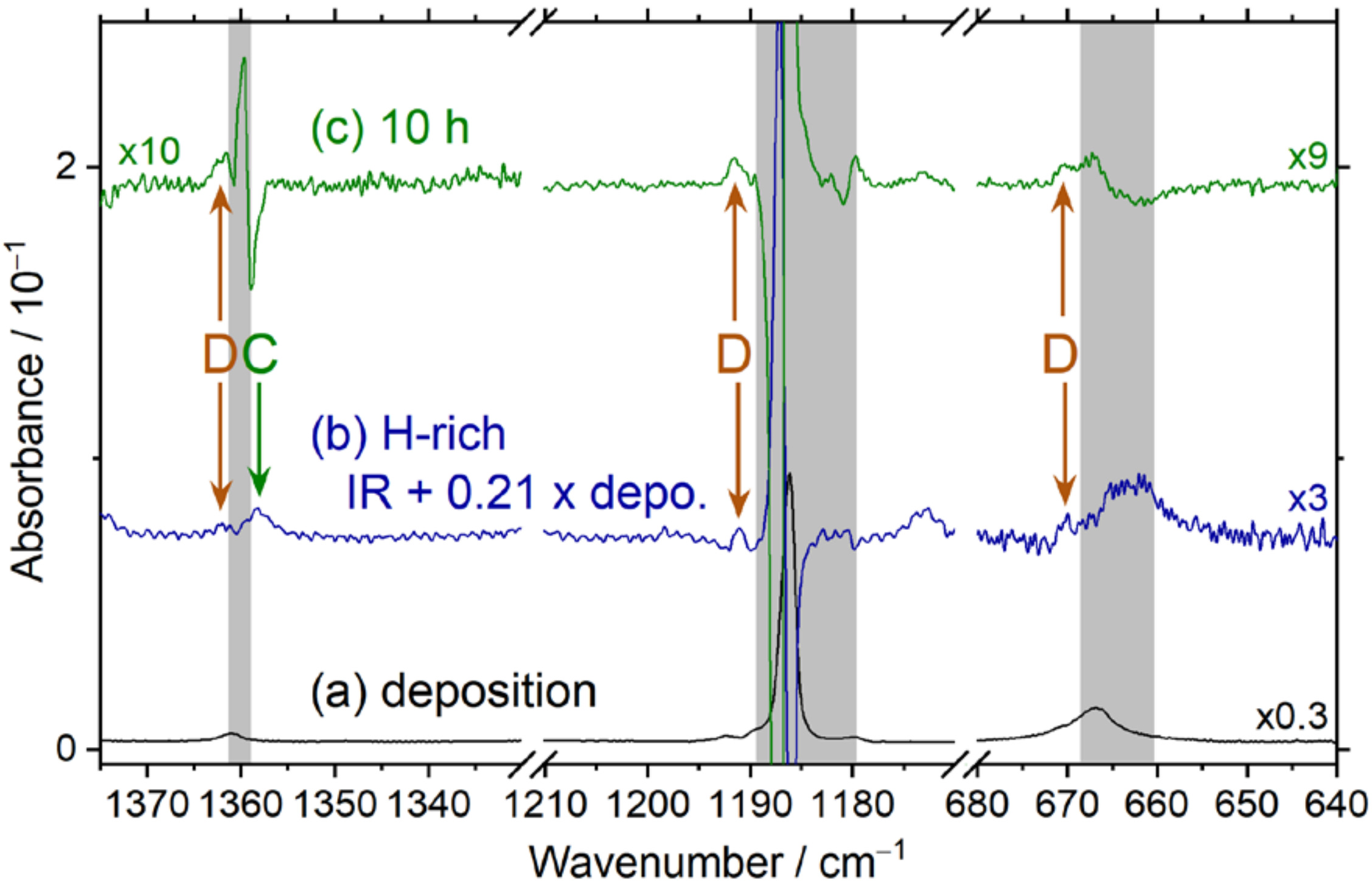

Figure S19. Representative spectra for the identification of bands in group D in a uracil/Cl_2/*p*-H_2 (1/20/10000) matrix. (a) Spectrum recorded after deposition at 3.2 K for 6 h. (b) Processed spectrum of the difference spectrum after IR irradiation, reproduced from the upper trace of Figure S15: Figure 3c + 0.21 × Figure 3a and Figure S9c + 0.21 × Figure S9a, obtained from the H-rich experiment with uracil/Cl_2/*p*-H_2 (1/20/10000). (c) Difference spectrum after the full IR irradiation and maintaining the matrix in darkness for 10 h, from Figures 3d and S9d. Bands in groups C and D are indicated with green and brown arrows and labels, respectively. The areas shaded in gray indicate spectral regions that are interfered with by uracil absorption.

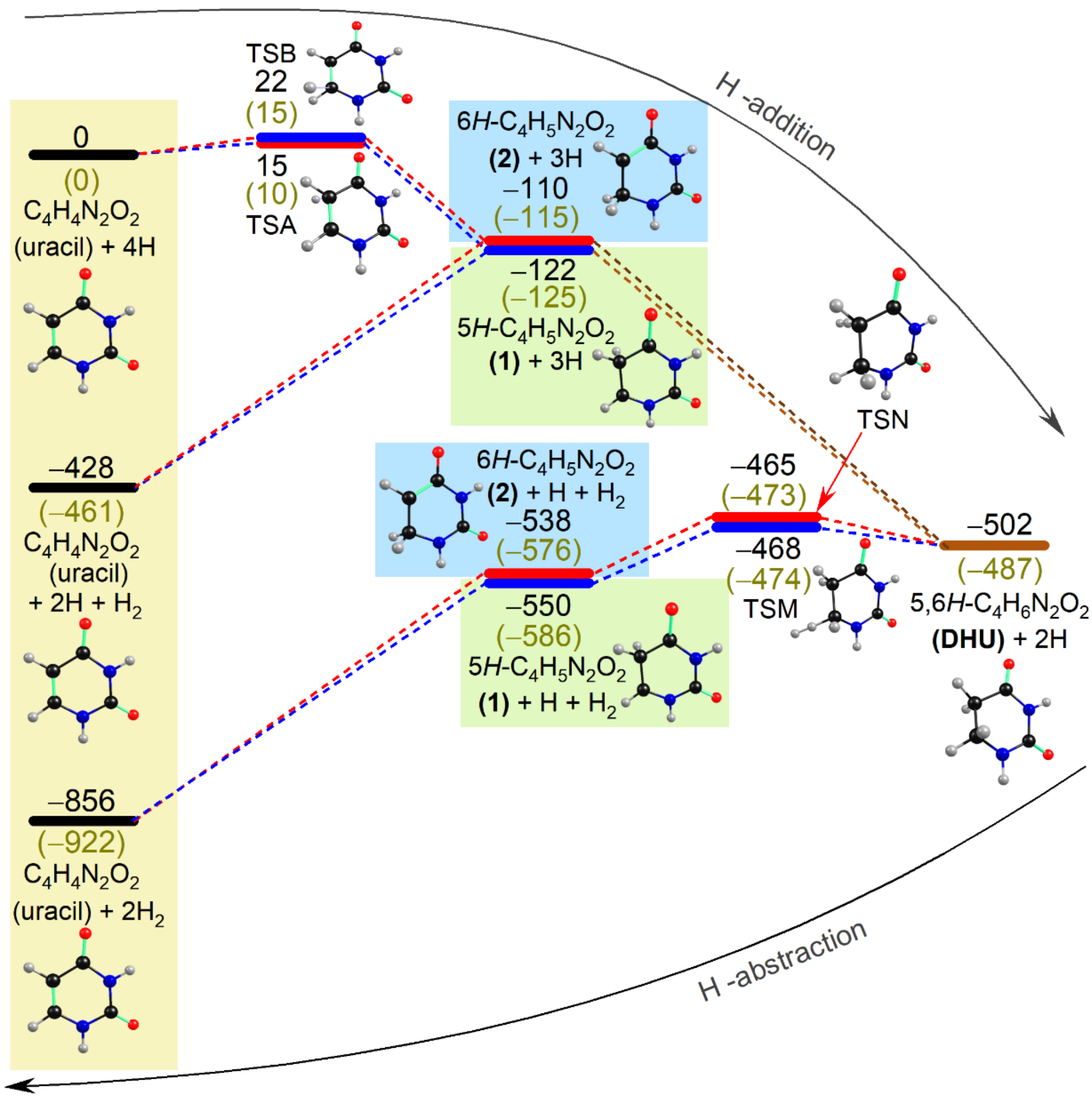

Figure S20. Potential-energy scheme predicted for the formation of 5,6-dihydrouracil (DHU) and regeneration of uracil in the reaction H + uracil. Hydrogen addition to uracil produces 5*H*-$C_4H_5N_2O_2$ **(1)** and 6*H*-$C_4H_5N_2O_2$ **(2)**; subsequent hydrogen addition to **(1)** or **(2)** produces DHU. Hydrogen abstraction of DHU produces **(1)** or **(2)**, which regenerates uracil via subsequent hydrogen abstraction. Energies (in kJ mol^{-1}) relative to 4H + uracil were calculated using the CCSD(T)/aug-cc-pVTZ//B3LYP/aug-cc-pVTZ method (black); values calculated using the B3LYP/aug-cc-pVTZ method are listed in parentheses (olive). Zero-point vibrational energies (ZPVE) from the B3LYP/aug-cc-pVTZ method were corrected.

Supplementary References

[1] Tam, S.; Fajardo, M. Single and Double Infrared Transitions in Rapid-Vapor-Deposited Parahydrogen Solids: Application to Sample Thickness Determination and Quantitative Infrared Absorption Spectroscopy. *Appl. Spectrosc.* **2001**, *55*, 1634−1644. DOI: 10.1366/0003702011953946

[2] Silvera, I. F. The Solid Molecular Hydrogens in the Condensed Phase: Fundamentals and Static Properties. *Rev. Mod. Phys.* **1980**, *52*, 393–452. DOI: 10.1103/RevModPhys.52.393

[3] Fajardo, M. E. Matrix Isolation Spectroscopy in Solid Parahydrogen: A Primer. In *Physics and Chemistry at Low Temperatures*, Khriachtchev, L., Ed.; Jenny Stanford Publishing, 2011. DOI: 10.1201/9780429066276-6
